# Occurrence of 3-nitrobenzanthrone and other powerful mutagenic polycyclic aromatic compounds in living organisms: polychaetes

**DOI:** 10.1038/s41598-020-60369-9

**Published:** 2020-02-26

**Authors:** Maria Claudia R. Sola, Aldenor G. Santos, Sabrina T. Martinez, Madson M. Nascimento, Gisele O. da Rocha, Jailson B. de Andrade

**Affiliations:** 1 Instituto Nacional de Ciência e Tecnologia em Energia e Ambiente - INCT, Universidade Federal da Bahia, 40170-115 Salvador, BA Brazil; 2 Centro Interdisciplinar em Energia e Ambiente - CIEnAm, Universidade Federal da Bahia, 40170-115 Salvador, BA Brazil; 3 Instituto de Química, Universidade Federal da Bahia, Campus de Ondina, 40170-115 Salvador, BA Brazil; 4 Centro Universitário SENAI-CIMATEC, 41650-110 Salvador, BA Brazil

**Keywords:** Environmental sciences, Chemistry

## Abstract

In this work we report the occurrence of powerful mutagenic 3-nitrobenzanthrone (3-NBA), in addition to 18 polycyclic aromatic hydrocarbons (PAHs), 6 oxygenated PAHs and 27 nitrated PAHs in polychaete worms. Benzanthrone (BA), another important mutagenic polycyclic aromatic compound (PAC) also was detected in the samples. Polychaete annelids have great ecological relevance, being widely distributed in different environmental conditions, from intertidal zones up to seven thousand feet deep areas. They are abundantly found in both contaminated and uncontaminated areas and, therefore, used as indicators of the pollution status of a given area. As we know, so far, most of these PACs has not been previously reported in living organisms before. The 3-NBA concentrations determined in this study were within 0.11–5.18 µg g^−1^. Other relevant PACs such as PAHs, quinones and nitro-PAHs were found in maximum concentrations at 0.013 µg g^−1^ (coronene) to 11.1 µg g^−1^ (benzo[k]fluoranthene), 0.823 µg g^−1^ (9,10-phenenthrenequinone) to 12.1 µg g^−1^ (1,4-benzoquinone) and 0.434 (1-nitronaphthalene) µg g^−1^ to 19.2 µg g^−1^ (6-nitrobenzo[a]pyrene), respectively. Principal component analysis (PCA), ternary correlations and diagnostic ratios were employed in order to propose probable sources for PACs. Although statistical analysis preliminarily has indicated both pyrogenic and petrogenic contributions, petrogenic sources were predominant reflecting the impacts of petroleum exploration and intensive traffic of boats in the study area.

## Introduction

Within the last several decades, estuarine and coastal environments (principally those in the vicinities of urbanized, industrialized, and/or populated areas) have been heavily impacted or have had the pollution status aggravated by a variety of anthropogenically-emitted organic and inorganic pollutants^1–7^. Coastal and estuarine ecosystems are complex systems subjected to significant limitations in physical and chemical processes (e.g., tides, freshwater intakes, temperature variations, among others) and may also be exposed to high contaminant concentrations. Many persistent chemical pollutants such as polycyclic aromatic compounds (PACs) are deposited in these ecosystems where they may cause damage to the benthic environment and are likely to become biomagnified through the food chain, ultimately posing risks to human health^8^.

Due to the inherent hydrophobicity and lipophilicity of some PACs such as polycyclic aromatic hydrocarbons (PAHs), once they are released into water bodies they tend to be primarily bound to suspended particles in the water column, and are eventually deposited on the seafloor in sediments and particles where they may be rapidly accumulated by marine benthic invertebrates such as polychaetes worms. Hence, marine polychaetes are well known for their PAH metabolism capabilities, although the ability to metabolize PAHs may widely vary among families^1,2,4,9–20^.

In October 1997 the first paper was published registering the isolation of 3-nitrobenzanthrone (3-NBA) from the organic extract of both diesel exhaust and airborne particles. 3-NBA was identified as a new class of powerful direct mutagen and was called “the devil in the diesel”^21^. Due to it has induced the highest number of revertants per nanomole in the Ames test to date. In this way, 3-NBA, a nitroketone polycyclic aromatic compound, has attracted much attention from the scientific community due to its genotoxic and mutagenic potential even if present in extremely small quantities in diesel exhaust particles^22–26^.

Although the occurrence of traditional PAHs and other petroleum-related substances in abiotic compartments of marine environments have been subject of several studies^6,27–30^, much less attention has been paid to the effects of these substances on living organisms, which some of them may be the base of the marine food chain (i.e. polychaetes). Consequently, little is also known about the possible endpoints of these pollutants may induce in different trophic levels. Polychaetes are likely to be resistant to high levels of organic pollutants in marine environments^4,11,31–34^. For instance, it is commonly cited the opportunism of the genus *Capitella* due to the ability to remediate sediments contaminated with organic pollutants^18^.

The main objective of the present study was to investigate the occurrence of “unconventional” PACs in polychaetes. Thus, we determined the concentrations of 3-NBA and 18 PAHs, 27 nitro-PAHs and 6 oxy-PAHs in polychaete samples from different families. Traditional sample preparation procedures for chromatographic analysis of marine living organisms may involve the use of Soxhlet extraction or other traditional techniques, which involve additional laborious and time-consuming clean-up and fractionating steps. Thus, an alternative and very effective sample preparation method for solid-liquid microextraction of polychaetes organisms was employed, which involves the direct extraction using masses as lower as 8 to 200 mg of polychaetes and 500 µL of the extraction solvent (18% acetonitrile in dichloromethane). Multivariate statistical analysis and binary and ternary correlations were performed to better understand the occurrence of these compounds in living organisms. To date, this is the first time that 3-NBA, amongst a comprehensive list of PAHs, quinones, and nitro-PAHs, are reported in polychaetes collected from a marine area.

## Results and Discussion

### Chromatographic analysis and identification of PACs

In our previous works^35,36^ we developed a chromatographic method for simultaneous determination of PACs, including PAHs, nitro-PAHs and quinones associated to atmospheric particulate matter by gas chromatography coupled to mass spectrometry (GC-MS), demonstrating that 3-NBA is emitted directly from vehicles and diesel combustion and that 2-NBA is formed during aerosol transport into the atmosphere. In the present work, we focused on the relationships among 3-NBA, benzanthrone (BA), and related PAHs, quinones and nitro-PAHs in polychaete worms. The 3-NBA, BA and other PACs were identified based on both its retention time and mass spectrum, which were acquired in SCAN mode. Furthermore, BA was well-separated from 3-NBA (Fig. S1A,B) and other PACs in the applied chromatographic conditions (Figs. S2–S5).

In order to approach the unequivocal identification of BA and 3-NBA, we monitored two m/z ions, i.e., the ion base and the reference ions for each compound (Table S1). In this way, BA was identified by using m/z 230 (ion base) and m/z 202 (Fig. S1C). In turn, 3-NBA (Fig. S1D) was identified by m/z 275 (base ion) and m/z 245 (reference ions). Quantification was carried out by further considering the ion base signal only, in SIM mode.

### Occurrence of polycyclic aromatic compounds in polychaetes collected in the Todos os Santos Bay (BTS) region

As shown in Table 1, low-molecular-weight (LMW) PAHs such as naphthalene (NAP), acenaphthylene (ACY), acenaphthene (ACE), fluorene (FLU), phenanthrene (PHE), and anthracene (ANT) were detected in all analyzed samples with maximum concentrations ranging from 0.800 µg g^−1^ dry weight (dw) (ANT) to 7.14 µg g^−1^ dw (PHE). These values were similar to those presented by Szczybelski *et al*.^37^ which reported LMW PAH concentrations in the range of 0.149 µg g^−1^ dw (ANT) to 2.46 µg g^−1^ (PHE) and 0.100 µg g^−1^ dw (ANT) to 2.28 µg g^−1^ dw (PHE) for polychaetes *Nephtys ciliata* and *Alitta virens*, respectively. In that study the authors analyzed the PAH content in polychaetes living in impacted sediment collected in Oosterchelde Estuary, Netherlands. In turn, high molecular weight (HMW) PAHs such as fluoranthene (FLT), pyrene (PYR), benzo(a)pyrene (BaP), and benzo(ghi)perylene (BgP) were detected in 82.8–100% samples in the present work. Although these compounds have been reported with a high frequency of detection, their maximum concentration range (0.013–0.600 µg g^−1^ dw) were lower than those observed for LMW PAHs, except for benzo(k)fluoranthene (BkF), which was found in concentrations above 11.0 µg g^−1^ dw. In a work published by Nesto *et al*.^38^, the concentrations of HMW PAHs such as PYR and benzo(a)anthracene (BaA) (ranging from 0.0066 to 0.039 µg g^−1^ dw, and from not detected to 59.3 µg g^−1^ dw, respectively) were determined in two lagoon sites in Venice, Italy. These sites were close to a system of navigable channels with intense shipping traffic and were in the vicinity of an industrial plant in the port of the city. These concentrations were very similar to the majority of samples collected in the sites from Todos os Santos Bay, BTS (from Portuguese, *Baía de Todos os Santos*, BTS, located in Northeastern Brazil) (median 0.003–0.033 µg g^−1^) but were lower than the maximum concentration of PYR (0.367 µg g^−1^ dw) and BaA (0.121 µg g^−1^ dw). The BTS sites were characterized by intense shipping and small boat traffic, waste discharge, and petroleum exploration that may have contributed to the concentrations of these organic pollutants found in polychaetes. Table 1 shows the PAC concentrations determined in this study and provides comparisons with other published data.

**Table 1:** Range of concentration (dry weight), frequency of detection for polycyclic aromatic compounds founds in the polychaete organisms collected in Todos os Santos Bay (BTS).

				Comparison with other published works			Uno *et al*.^41^	
This work				Szczybelski *et al*.^16^		Nesto *et al*.^38^		
PAHs	Median	Range	FOD (%)	Range (µg g^−1^)	*A*. virens*	Range (µg g^−1^)	Range (µg g^−1^)	
	(µg g^−1^ dw)	(µg g^−1^ dw)						
Compounds				*N*. ciliata*		*P*. rullieri*	Oysters	Mussels
(NAP)	0.243	0.020–5.52	100	—	—	—	—	—
(ACY)	0.094	0.005–1.91	100	—	—	—	—	—
(ACE)	0.147	<LOD–5.12	100	—	—	—	—	—
(FLU)	0.303	0.038–4.27	100	—	—	—	—	—
(PHE)	0.367	0.053–7.14	100	0.729–2.46	1.32–2.28	0.0095–0.029	—	—
(ANT)	0.042	0.030–0.800	100	0.149–0.336	0.100–0.113	—	—	—
(FLT)	0.033	<LOD-0.330	100	1.77–4.47	1.60–3.15	nd - 0.026	—	—
(PYR)	0.033	<LOD - 0.367	100	1.38–3.76	0.893–2.034	0.0066–0.039	—	—
(BaA)	0.003	<LOD - 0.121	68.9	0.077–0.218	0.083–0.134	59.26–0.00 nd - 0.059	—	—
(CRY)	0.002	<LOD - 0.196	48.3	1.5 × 10^−4^–6.43	n.d. - 0.887	<LOD - 0.0097	—	—
(BbF)	0.009	<LOD - 0.600	72.4	0.100–0.228	0.100–0.151	—	—	—
(BkF)	0.159	<LOD - 11.1	82.8	—	—	<LOD - 0.0033	—	—
(BaP)	0.040	<LOD - 0.543	82.8	—	—	<LOD - 0.0048	—	—
(PER)	0.023	<LOD - 0.254	65.5	—	—	—	—	—
(IND)	0.037	<LOD - 0.281	82.2	0.034 - n.d.	0.068–0.096	—	—	—
(DBA)	0.008	<LOD - 0.090	58.6	0.040–0.166	0.115–0.239	nd - 0.0043	—	—
(BgP)	0.067	<LOD - 0.446	96.6	0.344–1.106	n.d. - 0.134	<LOD - 0.029	—	—
(COR)	0.001	<LOD - 0.013	51.7	—	—	—	—	—
**Oxy-PAHs**		**Range (µg g**^**−1**^**)**	**FOD (%)**					
(1,4-BQ)	0.124	<LOD - 12.1	82.8	—		—	—	—
(1,2-NQ)	<LOD	—	93.1	—		—	—	—
(1,4-NQ)	0.162	<LOD - 4.57	89.7	—		—	—	—
(9,10-AQ)	0.221	0.019–2.20	93.1	—		—	—	—
(9,10-PQ)^AV^	0.084	0.099–0.823	44.8	—		—	—	—
(BA)	0.031	<LOD - 0.520	82.8	—		—	—	—
**Nitro-PAHs**		**Range (µg g**^**−1**^**)**	**FOD(%)**					
(1-NNAP)	0.064	<LOD - 0.434	100	—		—	3.23 × 10^−4^–4.78 × 10^**−**3^	4.38×10^**−**4^–7.63 × 10^**−**3^
(2-NNAP)	0.041	<LOD - 0.784	100	—		—	1.4 × 10^**−**5^–8.25 × 10^**−**3^	3.80 × 10^**−**4^–6.44 × 10^**−**3^
(1M-4NNAP)	0.038	0.010–0.888	82.6	—		—	—	—
(1M-5NNAP)	0.184	0.014–12.24	93.1	—		—	—	—
(1M-6NNAP)	0.153	0.017–2.50	96.6	—		—	—	—
(2M-4NNAP)	0.203	0.020–2.84	86.2	—		—	—	—
(2-NBP)	0.236	0.022–3.06	100	—		—	—	—
(3-NBP)	0.084	0.011–2.14	89.7	—		—	—	—
(4-NBP)	0.009	0.009 - 0.77	51.7	—		—	—	—
(5-NACE)	0.126	0.019–1.31	96.6	—		—	—	—
(2-NFLU)	0.476	0.006–2.74	100	—		—	5.81 × 10^**−**5^–6.19 × 10^**−**4^	8.2 × 10^**−**5^–5.910^**−**4^
(2-NPHE)	0.101	0.008–0.790	93.1	—		—	—	—
(3-NPHE)^AV^	0.067	<LOD - 0.500	37.9	—		—	2.2 × 10^**−**5^–0.206	1.21 × 10^**−**4^ - 9.87 × 10^**−**3^
(9-NPHE)	0.041	0.007–0.650	72.4	—		—	6.83 × 10^**−**4^–0.179	4.85 × 10^**−**4^–6.69 × 10^**−**3^
(2-NANT)	<LOD	—	82.8	—		—	—	—
(9-NANT)	0.041	<LOD - 2.83	65.5	—		—	—	—
(2-NFLT)^AV^	<LOD	—	37.9	—		—	—	—
(3-NFLT)^AV^	0.518	0.112 - 4.52	44.8	—		—	—	—
(2-NPYR)^AV^	0.011	<LOD - 0.153	17.2	—		—	—	—
(4-NPYR)^AV^	<LOD	—	13.8	—		—	6.4 × 10^**−**5^–9.8 × 10^**−**4^	72 × 10^**−**5^–6.24 × 10^**−**4^
(7-NBaA)	<LOD	—	89.6	—		—	—	—
(6-NBaP)	1.715	0.296–19.2	96.6	—		—	—	—
(1-NBeP)	1.513	0.09–7.99	93.1	—		—	—	—
(3-NBeP)	1.140	0.517–11.30	79.3	—		—	—	—
(1-NPYR)	0.131	0.017–2.32	72.4	—		—	4.52 × 10^**−**5^–2.07 × 10^**−**4^	32 × 10^**−**5^–31 × 10^**−**4^
(6-NCRY)	0.133	0.026–2.83	75.9	—		—	2.868 × 10^**−**5^–7.47 × 10^**−**5^	1.21 × 10^**−**4^–2.10 × 10^**−**3^
(3-NBA)	0.505	0.110–5.18	100	—		—	—	—

A comparison with other works is performed.

Nephtys ciliata (Müller, 1788), Alitta virens (M. Sars, 1835), Perinereis rullieri Pilato, 1974,

*Polychaetes.

FOD, Frequency of detection.

^AV^Average.

dw, dry weight.

Nitronaphthalenes were detected in most samples (82.6–100%) with median values ranging from 0.064 µg g^−1^ to 0.203 µg g^−1^. However, their concentrations did not exceed 3.0 µg g^−1^ dw, except for 1-methyl-5nitronaphthalene (1M-5NNAP) which was present in concentrations up to 12.2 µg g^−1^ dw. The concentrations of these nitro-PAHs may be explained by the abilities of polychaetes to absorb and/or bioaccumulate many organic pollutants^4,17,20,39,40^. For instance, K. Ito *et al*.^40^ investigated the bioaccumulation capacity of the oligochaete *Thalassodrilides* sp. and the polychaete *Perinereis nuntia* for nitro-PAHs such as 1-nitronaphthalene (1-NNAP). Organisms were exposed to 1400 µg L^−1^ of 1-NNAP in seawater for three days in the dark at 20 °C. They observed an increase in the 1-NNAP content from 0.012 to 0.094 µg g^−1^ (an approximately 8x increase) in *Thalassodrilides* sp. and from 0.9 ng g^−1^ to 38 µg g^−1^ in *P*. *nuntia* (more than 42,000x increase), showing that *P*. *nuntia* has a higher ability to bioaccumulate 1-NNAP than *Thalassodrilides* sp. In our study, 1-NNAP and 2-nitronaphthalene (2-NNAP) were found at levels below those reported in *P*. *nuntia*^40^. However, it should be kept in mind that the study by K. Ito *et al*.^40^ involved experiments in controlled conditions whereas in our study different polycyclic compounds were determined in polychaetes specimens, mainly belonged to the Opheliidae, Capitellidae, Spionidae, Goniadidae, Syllidae, and Orbiniidae families, collected from the wild and directly analyzed without any exposure to chemical substances at the laboratory. Therefore, these studies are not directly comparable.

Regarding sampling of other organisms from marine environments, it is worth mentioning that 1-NNAP and 2-NNAP contents were also determined by Uno *et al*.^41^ in mussel and oyster samples collected from Osaka Bay. The concentrations in mussels ranged from 4.38 × 10^−4^ to 0.0076 µg g^−1^ and 3.95 × 10^−4^ to 0.0052 µg g^−1^ for 1-NNAP and 2-NNAP, respectively. In oysters, the 2-NNAP concentrations exceeded 0.018 µg g^−1^, which were very similar to the concentrations in most polychaete samples in this study. In addition, the concentrations of 3-nitrophenanthrene (3-NPHE) (0.206 µg g^−1^ dw) and 9-nitrophenanthrene (9-NPHE) (0.179 µg g^−1^ dw) found in oysters^41^ were the highest. However, they are lower than the maximum concentrations in polychaetes determined in this study, which ranged from 0.026 to 0.500 µg g^−1^ dw, and from 0.007 to 0.650 µg g^−1^ dw for 3-NPHE and 9-NPHE, respectively.

Nitrated PAHs (such as 1M-5NNAP, 1-nitrobenzo(e)pyrene (1-NBeP), 3-nitrobenzo(e)pyrene (3-NBeP), and 6-nitrobenzo(a)pyrene (6-NBaP)) were found in high concentrations in some samples collected from BTS. The median concentrations of these compounds ranged from 0.184 µg g^−1^ dw (1N-5NNAP) to 1.72 µg g^−1^ dw (6-NBAP). Other nitro-PAHs were found at detection frequencies higher to 72%, except for 4-nitrobiphenyl (4-NBP), 3-NPHE, 2-nitrofluoranthene (2-NFLT), 3-nitrofluoranthene (3-NFLT), 2-nitropyrene (2-NPYR), and 4-nitropyrene (4-NPYR) which had detection frequencies of 13.8‒51.7%. In addition, some oxy-PAHs (such as 1,4-benzoquinone (1,4-BQ), 1,2-naphthoquinone (1,2-NQ), 1,4-naphthoquinone (1,4-NQ), and 9,10-anthraquinone (9,10-AQ)) were detected at high frequencies (lower than 93%), although 1,2-NQ was below the limit of detection (LOD). The median concentrations of oxy-PAHs within the range of 0.031 µg g^−1^ dw (BA) to 0.221 µg g^−1^ dw (9,10-AQ). The oxy-PAH 9,10-phenanthraquinone (9,10-PQ) showed a low frequency of detection and the lowest concentration range compared to other oxy-PAHs (<LOD–0.823 µg g^−1^ dw).

It is notable that the potent mutagenic and carcinogenic compound 3-NBA was detected in all samples with concentrations ranging from 0.110 to 5.18 µg g^−1^ dw. Benzanthrone, another important mutagenic pollutant, was detected in 82.8% of samples with concentrations ranging from <LOD to 0.520 µg g^−1^ dw. As far as we know, there are no reports in the literature regarding 3-NBA and BA concentrations in marine organisms, and therefore, these concentrations were determined for the first time in polychaetes in this study. A chromatogram (SIM mode) of 3-NBA peak detected in a real sample is shown in Fig. S2.

To the best of our knowledge, there are no studies addressing diagnostic ratios, and their related reference values against different sources, for nitro- and oxy-PAHs in living organisms. Hence, it is difficult to accurately determine the main sources of these compounds that were found in polychaete samples by using diagnostic ratios. Alternatively, PCA and ternary correlations combined with diagnostic ratios calculations (for PAHs only) may enable the determination of major sources of nitro- and oxy- PAHs. PCA was employed in order to reduce the dataset dimensionality while preserving the majority of its statistical information. This technique is an efficient statistical tool that allows identification of samples containing relevant information in loadings and scores in bidimensional plots^42^. For reducing the number of variables, we divided our results according to the chemical classes and the number of rings in the chemical structure. In this way, we conveniently grouped our results into PAHs, oxy-PAHs, and nitro-PAHs. For each of them, we then grouped the PACs containing 2, 3, 4, 5, and 6 rings separately. For PAHs, we grouped those with 3 rings (ACE, ACY, FLU, PHE, and ANT), 4 rings (FLT, PYR, BaA, and chrysene (CRY)), 5 rings (BbF, benzo(k)fluoranthene (BkF), BaP, perylene (PER), and dibenzo(a,h)anthracene (DBA)), and 6 rings (indeno(123 cd)perylene (IND) and BgP). Similarly, we did the same for nitro-PAHs with 2 rings (1-NNAP, 2-NNAP, 1-methyl-4nitronaphthalene (1M-4-NNAP), 1M-5-NNAP, 1-methyl-6-nitronaphthalene (1M-6-NNAP), 2-methyl-4nitronaphthalene (2M-4-NNAP), 2-nitrobiphenyl (2-NBP), 3-nitrobiphenyl (3-NBP), and 4-NBP), 3 rings (5-nitroacenaphthene (5-NACE), 2-nitrofluorene (2-NFLU), 2-NPHE, 3-NPHE, 9-NPHE, 2-nitroanthracene (2-NANT), and 9-nitroanthracene (9-NANT)), 4 rings (2-NFLT, 3-NFLT, 2-NPYR, 4-NPYR, 7-nitrobenzo(a)anthracene (7-NBaA), 1-nitropyrene (1-NPYR), and 6-nitrochrysene (6-NCRY)), and 5 rings (6-nitrobenzo(a)pyrene (6-NBaP), 1-nitrobenzo(e)pyrene (1-NBeP), and 3-nitrobenzo(e)pyrene (3-NBeP)). This strategy was also employed by M. Ito *et al*.^4^, allowing a reduction of the number of variables without losing the ability of interpreting data. Thus, it was obtained a 29 × 17 data matrix (29 samples against 17 chemical species). Autoscaling was employed as an initial pretreatment to assure that all variables presented the same importance. A preliminary evaluation of the correlation matrix showed significant correlations for most of the variables. The criteria for the extraction of principal component (PCs) was based on eigenvalues ≥1 and the percentage of variance in each PC (Table 2). Please, also see Figs. S7 and S8.

**Table 2:** Rotated factor loadings of extracted principal components.

Variables	PC1	PC2	PC3	PC4	PC5
NAP	**0**.**847**	0.478	0.014	0.033	−0.142
3-Rings	0.264	**0**.**921**	0.154	−0.002	0.029
4-Rings	0.267	**0**.**746**	−0.176	0.026	0.249
5-Rings	**0**.**908**	0.270	−0.170	0.109	−0.144
6-Rings	**0**.**765**	0.332	−0.314	0.083	0.225
COR	0.036	−0.063	−0.166	−0.068	**0**.**900**
1,4-BQ	0.055	−0.019	0.061	**0**.**939**	−0.053
1,2-NQ	0.068	**0**.**954**	0.030	−0.030	−0.105
1,4-NQ	**0**.**845**	0.189	0.227	−0.134	0.282
9,10 -AQ	0.160	0.119	−**0**.**714**	−0.030	0.360
9,10-PQ	−0.168	−0.190	−0.609	−0.294	0.090
N-2 Rings	**0**.**928**	0.191	0.168	0.050	0.145
N-3 Rings	0.461	**0**.**866**	−0.043	0.016	0.017
N-4 Rings	0.306	**0**.**906**	−0.088	0.005	−0.055
N-5 Rings	**0**.**786**	0.157	0.021	0.129	0.267
3-NBA	**0**.**659**	0.070	0.374	−0.276	0.274
BA	**0**.**882**	0.268	−0.190	−0.010	−0.239
Total variance (%)	48.9	14.1	10.2	6.49	5.47
Cumulative (%)	48.9	62.9	73.1	79.6	85.1

As shown in Table 2, five PCs were extracted by explaining 85.1% of the cumulative variance in the dataset. The PC1 explained more than 48% of the variance and was characterized by high loadings of NAP and PAHs containing 5 and 6 rings, nitro-PAHs containing 2 and 5 rings, 1,4-NQ, 3-NBA, and BA. In turn, PC2, which explained 14.1% of the variance, was characterized by high loadings for 3- and 4-ring PAHs, 1,2-NQ, and 3- and 4-ring nitro-PAHs. The 3- and 4-ring PAHs were strongly correlated with their nitrated analogues (3 and 4-ring nitro-PAHs). The PC3 and PC4 were characterized by high loadings for quinones, and PC5 showed high loadings for COR. The distribution of variables and samples in bidimensional graphs (score and loadings) can be seen in Figs. S4 and S5, where also it is presented a brief discussion about sample discrimination.

These PCA results may suggest that chemical species grouped in a specific PC (species presenting high loadings in the same given PC) might be either formed, have undergone similar physical-chemical processes, and/or been absorbed in polychaetes in a similar way. For instance, in PC1, NAP presented a high loading along with 1,4-NQ and N-2 rings. This may be representative of different rates of polychaetes absorption of these species or even the oxy- and nitro-derivatives of NAP (1,4-NQ and NNAP, respectively). Similar behavior was also observed in PC2 for 3-and 4-ring PAHs and nitro-PAHs. However, this hypothesized process is not well known or well understood and therefore it should be better studied become accepted. Indeed, this kind of reasoning should be taken very cautiously, and this hypothesis should be comprehensively studied prior to it being considered probable. However, in PC1, there were high loadings of 3-NBA and BA. It has been reported that 3-NBA is directly emitted by incomplete combustion of fossil diesel^21,36,43–45^. Although we have not located studies reporting BA levels in the atmosphere, it may be reasonable to accept that BA and 3-NBA may be formed in the same process. However, this hypothesis should be further addressed in future studies. These hypotheses are somewhat feasible under specific environmental conditions, for example, BA and 3-NBA may be transferred from the atmosphere to water bodies. Even though PC3, PC4, and PC5 explained small portions of the dataset, they were statistically significant for 9,10-AQ, 1,4-BQ, and COR, respectively.

### Molecular diagnostic ratios

**Figure 1:**
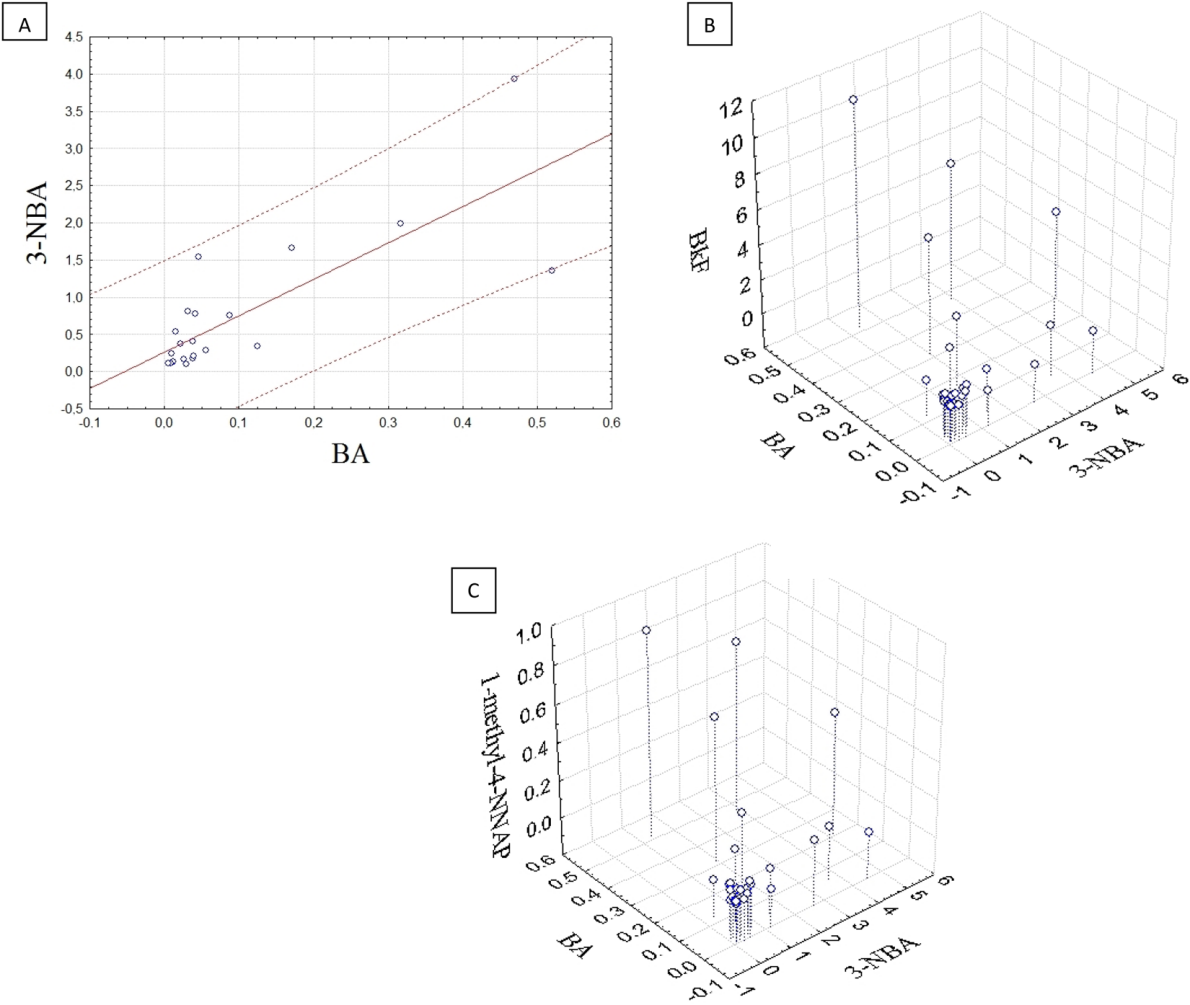
(**A**) Binary correlation plot between 3-NBA and BA (y = 0.2625 + 4.8938×; R = 0.7908; p < 0.0001); (**B**) 3D scatterplot of ternary correlation between BA and 3-NBA against BkF (R(z/xy) = 0.9474; p < 0.0001) and (**C**) 3D scatterplot of ternary correlation between BA and 3-NBA against 1-methyl-4-NNAP (R(z/xy) = 0.9667, p < 0.0001).

The use of PAH ratios has been widely applied to determine possible sources of contamination in the aquatic environment^46,47^. Although we are aware that diagnostic ratios should be used carefully since there is some inherent uncertainty associated with them, we chose to use the ratios involving PAH isomers with similar relative thermodynamic stabilities^48,49^ to avoid distortions in the source inferencing. The results showed that BA and 3-NBA were well correlated (r = 0.7908, p = 0.0001; Fig. 1), which supports the suggestion that they have close origins. In turn, compared to the other PACs, BA and 3-NBA presented strong ternary correlations (r > 0.75) (Fig. 1; Table 3) with 2-ring (NAP, 1-NNAP, 2-NNAP, 2-NBP, 3-NBP, 4-NBP, 1-methyl-6-NNAP, and 1-methyl-4-NNAP), 3-ring (ACE, 5-NACE, 2-NFLU, and 2-NPHE), 5-ring (BkF, PER, and 3-NBeP), and 6-ring PAHs and nitro-PAHs. Indeed, this is in good agreement with the chemical species found to have high loadings in PC1 (Table 2).

**Figure 2:**
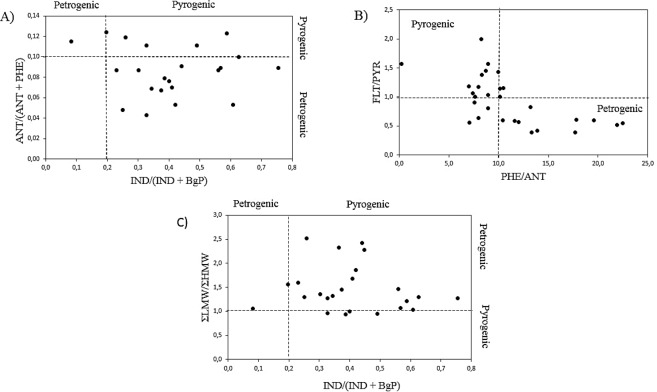
Selected molecular diagnostic PAH ratios in marine polychaetes: (**A**) ANT(ANT + PHE) versus IND(IND + BgP); (**B**) FLT/PYR versus PHE/ANT; and (**C**) ΣLMW/ΣHMW versus IND(IND + BgP).

**Table 3:** Binary and ternary correlations among BA, 3-NBA and selected PACs.

Compound	Ternary Correlations with BA and 3-NBA		Compound	Ternary Correlations with BA and 3-NBA	
	*r*-value	*p*-value		*r*-value	*p*-value
	0.9252	<0.0001		0.8273	<0.00001
	0.9447	<0.0001		0.9479	<0.0001
	0.7815	<0.00001		0.8702	<0.00001
	0.7655	0.00001		0.859	<0.00001
	0.8247	<0.00001		0.8852	<0.00001
	0.7638	0.00001		0.8339	<0.00001
	0.7626	0.00001		0.8465	<0.00001

Accordingly, we considered the following ratios: IND/(IND + BgP), ANT/(ANT + PHE), FLT/PYR, PHE/ANT, and ∑LMW/∑HMW disposed as cross plots (Fig. 2) within their respective reference values to discriminate pyrogenic from petrogenic contributions^46,47^.

Firstly, ANT/(ANT + PHE) > 0.10 indicated pyrogenic sources while ANT/(ANT + PHE) <0.10 indicated petrogenic inputs. Similarly, IND/(IND + BgP) <0.2 indicated petrogenic inputs, and >0.2 indicated pyrogenic inputs. Our data showed mixed contributions, with a predominance of petrogenic inputs. In general, petrogenic sources possess a higher predominance of PHE over ANT, and of PYR over FLT^46^. In this way, PHE/ANT >10 and FLT/PYR < 1.0 represented petrogenic contributions (Fig. 2B). In Fig. 2B, the data ranged from a small to a high predominance of PHE over ANT, and FLT/PYR values varied from approximately 0.5 to 2.0, indicating contributions from both pyrogenic and petrogenic sources. The data distribution in Fig. 2C showed ANT/(ANT + PHE) >0.1 e IND/(IND + Bg) <0.2 ratios, suggesting a significant contribution of petrogenic sources.

The LMW to HMW PAH ratios versus IND/(IND + BgP) is presented in Fig. 2C. According to Miguel-Gallo *et al*.^50^ pyrogenic (combustion) processes produce mainly HMW compounds (4–6 rings), whereas a higher proportion of LMW compounds (2–3 rings) is generally found in unburned petroleum. In this way, ∑LMW/∑HMW > 1.0 indicates petrogenic predominance over pyrogenic (∑LMW/∑HMW < 1.0) inputs. By calculation of the LMW/∑HMW ratios, our results show that the petrogenic contribution was prevalent.

For BTS, the contributing petrogenic sources may be the traffic of small boats or commercial ships (since two important harbors are located in this bay), petroleum exploitation, and complex petrochemical discharges. Pyrogenic sources appear to be fossil fuel combustion processes occurring in the vicinity of the BTS. It is likely the polycyclic levels found in polychaetes in the present study may be a result of petroleum exploitation in the area together to the transit of boats and other vessels around the bay. Additionally, PACs primarily emitted from automobiles to atmosphere and then transferred to water bodies might also be a contributing route explaining at least partially to the found PAC levels in BTS within the present study.

### Concluding remarks

For the first time it is reported the occurrence of potent mutagenic 3-NBA – together to other nitro- and oxy-PAHs - in polychaete organisms. The use of statistical analysis allowed the identification of possible sources of nitrated and oxygenated derivatives of HPAs. Contributing sources were petrogenic (from the traffic of small boats or commercial ships, petroleum exploitation and petrochemical complex discharges) as well as pyrogenic sources seem to be fossil fuel combustion processes occurring the vicinities of the bay.

## Methods

### Reagents and standards

Nitro-PAHs certified standards SRM 2264^51^ composed by 1-nitronaphthalene (1-NNAP), 2-nitronaphthalene (2-NNAP), 1-methyl-4-nitronaphthalene (1-methyl-4-NNAP), 1-methyl-5-nitronaphthalene (1-methyl-5-NNAP), 1-methyl-6-nitronaphthalene (1-methyl-6-NNAP), 2-methyl-4-nitronaphthalene (2-methyl-4-NNAP), 2-nitrobiphenyl (2-NBP), 3-nitrobiphenyl (3-NBP), 4-nitrobiphenyl (4-NBP), 5-nitroacenaphthene (5-NACE), and 2-nitrofluorene (2-NFLU) and SRM 2265^52^ (2-nitrophenanthrene (2-NPHE), 3-nitrophenanthrene (3-NPHE), 9-nitrophenanthrene (9-NPHE), 2-nitroanthracene (2-NANT), 9-nitroanthracene (9-NANT), 2-nitrofluoranthene (2-NFLT), 3-nitrofluoranthene (3-NFLT), 1-nitropyrene (1-NPYR), 2-nitropyrene (2-NPYR), 4-nitropyrene (4-NPYR), 6-nitrochrysene (6-NCRY), 7-nitrobenz[a]anthracene (7-NBaA), 3-nitrobenzanthrone (3-NBA), 6-nitrobenzo[a]pyrene (6-NBaP), 1-nitrobenzo[e]pyrene (1-NBeP), and 3 nitrobenzo[e]pyrene (3-NBeP) were purchased from NIST (USA). EPA 610 PAH mix (Supelco, USA) was also used in this study^35^. It was composed by acenaphthene (ACE), acenaphthylene (ACY), anthracene (ANT), benz[a]anthracene (BaA), benzo[a]pyrene (BaP), benzo[b]fluoranthene (BbF), benzo[ghi]perylene (BgP), benzo[k]fluoranthene (BkF), chrysene (CRY), dibenz[a,h]anthracene (DBA), fluoranthene (FLT), fluorene (FLU), indeno[1,2,3-d]pyrene (IND), naphthalene (NAP), phenanthrene (PHE), and pyrene (PYR), at 2000 µg mL^−1^ each, in methanol: methylene chloride (1:1). Individual standards of coronene (COR) at 50 µg mL^−1^ and perylene (PER) at 1000 µg mL^−1^ were also used. Quinones standards, composed by 1,4-benzoquinone (1,4-BQ) (98%), 9,10-phenanthraquinone (9,10-PQ) (95%) and 9,10-anthraquinone (9,10-AQ) (99.4%) were purchased from Sigma–Aldrich (St. Louis, USA). Additionally, 1,2-naphthoquinone (1,2-NQ) (90%) and 1,4-naphthoquinone (1,4-NQ) (96.5%) were purchased from Fluka (St. Louis, USA). A 500 µg mL^−1^ quinone mix stock solution was prepared by dissolving the standards in tetrahydrofuran^35^ (THF) (J. T. Baker, USA). Benzanthrone (BA CAS# 82-05-3) (>98%) was acquired from TCI (Toshima, Tokyo). In this study, stock and analytical solutions were prepared by successive dilutions in acetonitrile (ACN) (chromatographic and spectroscopic grade, J.T. Baker, USA).

### Sampling site description

Samples were collected in the area of Todos os Santos Bay (BTS), located in Northwestern Brazil (13°S and 38°W)^53^. This is the second-largest bay in Brazil with an area of approximately 1233 km². Despite being located in an industrialized area, more than 60% of the perimeter of this bay is surrounded by sandy beaches, rocky shores, reefs, and mangroves. There is a petrochemical industry complex, and more than 3 million inhabitants are distributed between 15 cities^53^. Petroleum exploitation has been legally conducted since 1938 in this area (which was the first location in Brazil to have petroleum extracted) and the marine environment is likely to have suffered from this activity to some degree since that time^54^. Even though petroleum exploitation in BTS has substantially declined since then, the Brazilian Petroleum Agency reported that 2,809,948 m^3^ conventional petroleum was produced in 2017^55^. Despite the fact that petroleum extraction in BTS has historically been conducted for more than 80 years, studies reporting the possible deleterious effects of this practice on the marine environment or on living organisms are scarce^53^.

### Sampling of polychaetes

Four sites within the BTS, Northeastern Brazil, were selected for collection of organisms: Madre de Deus (12 °44′27″S, 38 °37′15″W), Inema (12 °48′38.3″S, 38 °29′39.7″W), Aratu (12 °47′36.9″S, 38 °29′14.5″W) and Ribeira (12 °54′34.2″S, 38 °29′51.8″W). At each site, three 15-m transects were established that were perpendicular to the shoreline, from the high tide mark, and were arranged along the intertidal zone. In each transect, three random faunal samples were collected using a stainless-steel sieve (15 × 15 cm), allowing the application of same sampling effort by beach width. The polychaete worms were transferred individually to an aluminum-foil recipient containing seawater. The recipient containing the samples was closed using aluminum foil and then transported to the laboratory using a cooled box. The samples were stored in the freezer at −21 °C. The investigated polychaete specimens showed that Opheliidae, Capitellidae, Spionidae, Goniadidae, Syllidae, and Orbiniidae were the most numerous families in the assessed areas. The PACs were determined in 29 polychaetes samples collected from the four sites around BTS.

### Instrumentation and chromatographic analysis

In this work, we used the chromatographic conditions previously developed by Santos *et al*.^35^. In brief, a gas chromatograph coupled to a mass spectrometer GC-MS QP2010Ultra (Shimadzu, Japan), equipped with an AOC-20i autosampler and split/splitless injector operating in splitless mode at 310 °C and purge time of 0.80 min was employed for PACs analysis. The injection volume was 1.00 µL. The chromatographic separation was performed using a Rtx-5MS gas capillary column (5% diphenyl, 95% dimethylpolysiloxane, 30 m × 0.250 mm ID × 0.25 µm of film thickness) (Restek, Bellefonte, USA). High purity helium (99.9999%) (White Martins, Brazil) was used as carrier gas under flow rate of 1.00 mL min^−1^. Oven temperature programing initiated at 70 °C (2 min), then rising from 70–200 °C (30 °C min^−1^, 5 min), and 200–330 °C (5 °C min^−1^, 0.67 min). Injector temperature was set at 310 °C and transfer line was 280 °C. Analysis was carried out at electron impact mode (EI) (70 eV). In order to approach unequivocal peak identification and increase the sensitivity, the SIM (Selected Ion Monitoring) mode was employed and three specific ions were chosen for each compound. However, for quantification we only used the most intense ion (base ion). The same chromatographic conditions have been used successfully in the study of PAC associated to atmospheric aerosols^35^.

### Sample treatment and miniaturized solid-liquid ultrasound-assisted extraction

**Figure 3:**
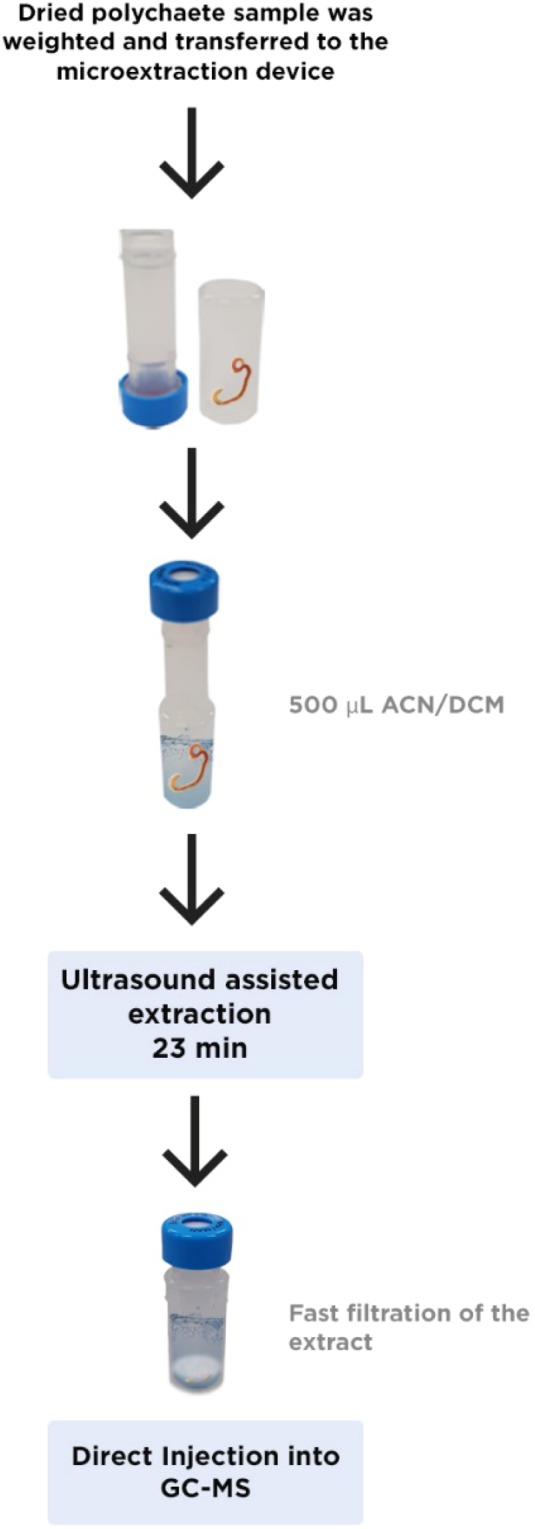
Schematic representation of the extraction procedure.

Polychaete organisms were thawed in room temperature and then, they were transferred to a desiccator, being kept by 24 h. For extraction of PACs from polychaetes, we employed a miniaturized solid-liquid extraction procedure the extraction procedure, previously developed by Santos *et al*.^35^ for extraction of PACs in PM2.5 samples. The procedure consisted of the use of a microextraction device Whatman MiniUniprep, composed by a polyethylene chamber and a plunger containing a PVDF filtering membrane. The whole device assumes the chromatography vial dimensions and it can be placed in the GC-MS autosampler for direct injection. This procedure was useful once allowed that small masses (8–100 mg) obtained from polychaetes could be accurately weighted and extracted. Briefly, the polychaete samples were directly weighted into polyethylene chamber of microextraction device and 500 µL of the extraction solvent (18% acetonitrile in dichloromethane) was added. The chamber was capped with the plunger and the extraction was carried out under sonication for 23 minutes. After that, the samples were filtered in the same microextraction device and then, they were directly injected in a GC-MS system. Figure 3 shows details of whole extraction procedure. The concentration was expressed in dry weight.

### QA/QC and method validation

All glassware and material used in the analysis were cleaned according EPA Method 610 for PAHs analysis (EPA, 1984). Instrumental, solvent and/or reagent and method blanks were checked for interfering compounds. The instrumental blank was assessed by analysis of carrier gas of GC-MS in Selected Ion Monitoring (SIM) mode. Solvent and/or reagent blank was checked by extraction of solvent mixture (18% ACN/DCM) using the microextraction device in absence of any sample. For method blank evaluation, a blank sample was prepared by extracting 100 mg of dried and powdered polychaete three times using 20 mL of 18% ACN/DCM mixture. The organic fraction was discarded, and the resultant mass was dried in a desiccator by 24 h. After that, approximately 5 mg pre-extracted sample was weighted and extracted following the abovementioned microextraction procedure. For instrumental and solvent and/or reagent blanks were not detected interfering compounds eluting in the same retention time of target compounds. Interfering peaks corresponding to naphthalene (0.87 pg <LOD), acenaphthene (0.31 pg <LOD), fluorene (1.09 pg <LOD), phenanthrene (4.01 pg <LOQ) and anthracene (0.09 pg <LOD) were detected in the method blank. However, these concentrations were discounted from samples.

Limits of detection (LOD) and limit of quantification (LOQ) were obtained from calibration curves parameters. We considered LOD = 3 s/a and LOQ = 10 s/a, where “s” is the standard deviation of the linear coefficient (b), and “a” is the angular coefficient (inclination) from calibration curve^56^. LOD and LOQ concentrations values were converted to the minimum absolute mass either detected (LOD) or quantified (LOQ) by the GC-MS in 1.00 µL of injected standard solution. For PAHs, nitro-PAHs and oxy-PAHs, the LOD in terms of absolute mass ranged from 0.83 pg (FLT) to 3.13 pg (PER), 0.81 pg (2-NFLU) to 15.2 pg (3-NPHE), and 0.58 (9,10-AQ) to 50 (1,2-NQ), respectively. Limit of quantification (LOQ) ranged from 2.75 pg to 10.4 pg, 2.70 pg to 50.7 pg, and 1.95 pg to 432 pg for these same compounds. In order to assess the extraction efficiency were performed recoveries test adding known concentrations of the mix standard solution of PACs to blank polychaete sample (Table S1). The recovery values for PAHs, nitro-PAHS and oxy-PAHs ranged from 87.6% (COR) to 114% (BaP) (RSD < 11.4%), 70.6% (6-NBaP) to 119% (5-NACE) (RSD < 11.6%), 94.3% (1,4-BQ) to 145 (1,4-NQ) (RSD < 11.4%) (Table S1). The RSD for the instrumental precision of this chromatographic method ranged from 0.47% (fluorene) to 5.11% (1,4-benzoquinone)^35^. Surrogate deuterated standards fluorene-d10 and pyrene-d10 were added in all sample and blanks before extraction. The recoveries of fluorene-d10 in all samples ranged from 103% to 132% with average and RSD of 117 ± 12% and pyrene-d10 showed recoveries ranging from 130 to 147% with average values of 138 ± 6.4%.

For identification and confirmation of 3-NBA, we re-analyzed suspect samples and then the same samples were spiked with a NIST 2265 standard of nitro-PAHs. After overlapping the chromatogram and mass spectra obtained from SIM mode, the presence of 3-NBA was confirmed in all suspected samples. As shown in the Fig. S6, all analyzed peaks were overlapped in the same retention time. The small differences between retention times of some samples can be attributed to the change of column during analyses. The mass spectra in SIM mode obtained from fragmentation of the 3-NBA show the presence of characteristic ions m/z 275 and 245.

### Statistical analysis

Principal component analysis (PCA) and ternary correlations were employed in order to simplify the dataset and to assess significant correlations between different variables (PACs). The statistical analysis was carried out using the software Statistica 7.0 (Tulsa, Oklahoma, USA). The Microsoft Excel datasheet (Microsoft, USA) was used for descriptive statistics and basic mathematical operations.

## Supplementary information

Supplementary info.
