# Supplementary material for: Occurrence of 3-nitrobenzanthrone and other powerful mutagenic polycyclic aromatic compounds in living organisms: polychaetes
Source: Sci Rep. 2020 Feb 26;10:3465. doi: 10.1038/s41598-020-60369-9 (PMC7044212; doi:10.1038/s41598-020-60369-9)
Supplement: Supplementary file 1 — Supplementary info. [file 41598_2020_60369_MOESM1_ESM.doc]

**Supplementary Information**

# Occurrence of 3-nitrobenzanthrone and other powerful mutagenic polycyclic aromatic compounds in living organisms: polychaetes

# Maria Claudia R. Sola^1, 2^, Aldenor G. Santos^1, 2, 3^, Sabrina T. Martinez^1, 2^, Madson M. Nascimento^2,3^, Gisele O. da Rocha^1, 2, 3^, Jailson B. de Andrade*^1, 2, 4^

^1^Instituto Nacional de Ciência e Tecnologia em Energia e Ambiente - INCT, Universidade Federal da Bahia, 40170-115 Salvador, BA, Brazil.

^2^Centro Interdisciplinar em Energia e Ambiente - CIEnAm, Universidade Federal da Bahia, 40170-115 Salvador, BA, Brazil.

_3_Instituto de Química, Universidade Federal da Bahia, Campus de Ondina, 40170-115, Salvador, BA, Brazil.

^4^Centro Universitário SENAI-CIMATEC, 41650-110, Salvador, BA, Brazil.

*Corresponding author: jailsondeandrade@gmail.com

**CAPTIONS**

Table S1. Chromatographic parameters and analytical features of the method.

Figure S1. A) GC-MS chromatogram in Scan mode showing the well-defined peak of 3-NBA eluting in 21.58 min and B) BA eluting in 21.58 min. C) Mass spectrum obtaining from 3-NBA fragmentation in electron impact mode (EI). D) BA mass spectrum showing characteristic ions *m/z* 230 (ion base), 202, 200 and 101 (reference ions).

Figure S2. General chromatogram in SIM mode of a real polychaete sample collected in Madre de Deus site. Each compound is shown in detail in Figures S4 and S5.

Figure S3. Real sample SIM chromatogram of detected PAHs and quinones in the Madre de Deus site. The compound were NAP (TR: 5.516 min), ACY (TR: 6.966 min), ACE (TR: 7.142 min), FLU (TR: 7.798 min), PHE (TR: 9.621 min), ANT (TR: 9.762 min), FLT (TR: 14.045 min), PYR (TR: 15.014 min), CRY (TR: 20.952 min), BkF (TR: 25.803 min), IND (TR: 31.113 min) and 9,10-AQ (TR: 12.427 min).

Figure S4. Chromatogram of nitro-PAHs detected in Madre de Deus sample. The compound were 1-NNAP (TR: 8.057 min), 2-NNAP (TR: 8.320 min), 1M-5NNAP (TR: 8.889 min), 2 M-4NNAP (TR: 9.700 min), 5-NACE (TR: 13.216 min), 9-PHE (TR: 17.190 min), 9-NANT (TR: 18.539 min), 7-NBaA (TR: 26.333 min), 6-NCRY (TR: 26.713 min), 3-NBA (TR: 27.61 min), 6-NBaP (TR: 31.731 min) and 3-NBeP (TR: 33.498 min).

Figure S5. A) GC-MS standard solution chromatogram acquired in SIM mode with the 3-NBA peak. B) Chromatogram in SIM mode showing 3-NBA peak detected in a polychaete sample. C) Mass spectrum of the 3-NBA in the same standard solution showing the characteristic ions *m/z* 275 (M+ and base ion) and *m/z* 245. D) Mass spectrum in SIM mode of 3-NBA detected in a real sample collected in the BTS site.

Figure S6. Comparison of different chromatogram for 3-NBA detected in different samples and spiked samples. The peaks were overlapped in order to facilitate the visualization of different samples.

Figure S7. Score graphs obtained from principal component analysis. A) PC1xPC2 with approximately 63 % of total variance, followed by B) PC1x`PC3, 59 %, C) PC1xPC4, 55 % and D) PC1xPC5, 54 %.

Figure S8. Loading graphs obtained from principal component analysis showing the distribution of polycheaetes samples. A) PC1xPC2 explaining approximately 63 % of total variance, followed by B) PC1x`PC3, 59 %, C) PC1xPC4, 55 % and D) PC1xPC5, 54 %. As can be seen in the loading graphs in Figure 1, the samples I_M_S3E1, MD_M_S1E1, A_M_S1E1 and A_J_S3E1 were discriminated in PC1 due to their high concentrations of naphthalene, 5 and 6-rings PAHs, nitro-PAHs containing 2 and 5 rings and oxy-PAHs such as 1,4-NQ and BA. As 3-NBA also presented high loading in this PC, these samples were characterized by high content of this compound. It is worth mentioning that these samples were collected throughout BTS sites corresponding to Inema, Madre de Deus, and Aratu sites. The samples collected in the Ribeira site (R_J_S2E2 and R_J_S3E2) were separated in PC2 and characterized by high concentrations of PAHs and nitro-PAHs containing 3 and 4 rings and oxy-PAHs such as 1,2-NQ. Although very little information is extracted from the last PCs, some oxy-PAHs such as 1,4-BQ and 9,10-AQ, and high molecular weight PAH like coronene (predominant in this PCs) were found in samples from Ribeira (R_J_S3E1, R_J_S2E2, R_M_S1E2, and R_M_S2E1), Aratu (A_M_S1E2, A_M_S1E1, and A_J_S3E1), and Inema (I_M_S3E1).

Table S1. Chromatographic parameters and analytical features of the method.

|  | SIM method parameters | | | Linearity | |  |  | Recovery test | |  |
| --- | --- | --- | --- | --- | --- | --- | --- | --- | --- | --- |
| PAHs | Ion# 1* | Ion #2** | RT |  |  | LOD | LOQ | Added 30 ng mL^-1^ |  |  |
|  | (m/z) | (m/z) | (min) | Linear range  (ng mL^-1^) | R² | (pg) | (pg) | Recovered  (ng mL^-1^)  (n=3) | Recovery (%)  (n=3) | RSD(%) (n=3) |
| (NAP) | 128 | 102 | 5.528 | 1.38 - 100 | 0.9993 | 1.38 | 4.59 | 30.9 ± 2.30 | 103 | 7.43 |
| (ACY) | 152 | 76 | 6.985 | 0.86 - 100 | 0.9996 | 0.86 | 2.86 | 28.5 ± 1.70 | 95.0 | 5.97 |
| (ACE) | 153 | 76 | 7.165 | 0.84 - 100 | 0.9997 | 0.84 | 2.80 | 33.1 ± 1.43 | 110 | 4.31 |
| (FLU-d10) | 176 | - | 7.771 | 1.12 - 100 | 0.9993 | - | - | - | - | - |
| (FLU) | 166 | 82 | 7.813 | 1.21 - 65 | 0.9988 | 1.21 | 4.01 | 32.6 ± 1.52 | 109 | 4.66 |
| (PHE) | 178 | 152 | 9.645 | 1.33 - 65 | 0.9989 | 1.33 | 4.42 | 31.7 ± 1.58 | 106 | 4.98 |
| (ANT) | 178 | 89 | 9.792 | 1.74 - 100 | 0.9987 | 1.74 | 5.78 | 29.8 ± 1.00 | 99.4 | 3.35 |
| (FLT) | 202 | 101 | 14.061 | 0.83 - 65 | 0.9994 | 0.83 | 2.75 | 28.8 ± 0.80 | 95.9 | 2.78 |
| (PYR-d10) | 212 | - | 14.940 | 1.12 - 65 | 0.9990 | - |  | - | - | - |
| (PYR) | 202 | 101 | 15.020 | 1.08 - 65 | 0.9990 | 1.08 | 3.60 | 28.0 ± 0.82 | 93 | 2.93 |
| (BaA) | 228 | 114 | 20.807 | 0.87 - 100 | 0.9995 | 0.87 | 2.90 | 31.5 ± 1.22 | 105 | 3.86 |
| (CRY) | 228 | 113 | 20.952 | 2.44 - 100 | 0.9979 | 2.44 | 8.12 | 27.9 ± 0.69 | 93 | 2.47 |
| (BbF) | 252 | 126 | 25.621 | 2.49 - 100 | 0.9978 | 2.49 | 8.29 | 31.4 ± 0.84 | 105 | 2.66 |
| (BkF) | 252 | 126 | 25.748 | 2.31 - 100 | 0.9981 | 2.31 | 7.68 | 31.9 ± 0.57 | 106 | 1.79 |
| (BaP) | 252 | 126 | 26.911 | 2.41 - 100 | 0.9979 | 2.41 | 8.02 | 34.2 ± 1.22 | 114 | 3.58 |
| (PER) | 252 | - | 27.245 | 3.13 - 100 | 0.9965 | 3.13 | 10.4 | 30.1 ± 2.06 | 100 | 1.69 |
| (IND) | 276 | 138 | 31.095 | 1.61 - 100 | 0.9991 | 1.61 | 5.37 | 30.8 ± 2.00 | 103 | 6.67 |
| (DBA) | 278 | 139 | 31.278 | 2.16 - 100 | 0.9983 | 2.16 | 7.19 | 29.6 ± 1.61 | 98.7 | 6.75 |
| (BgP) | 276 | 138 | 31.911 | 1.53 -100 | 0.9993 | 1.53 | 5.11 | 29.3 ± 1.60 | 97.8 | 5.49 |
| (COR) | 300 | - | 36.555 | 1.56 - 100 | 0.9989 | 1.56 | 5.20 | 26.3 ± 1.75 | 87.6 | 6.65 |
|  |  |  |  |  |  |  |  |  |  |  |
| Oxy-PAHs | Ion# 1* | Ion #2** |  |  |  | LOD | LOQ | Added 50 ng mL^-1^ |  |  |
|  | (m/z) | (m/z) | (min) | Linear range  (ng mL^-1^) | R² | (pg) | (pg) | Recovered (ng mL^-1^) | Rec (%)  (n=3) | RSD(%) (n=3) |
| (1,4-BQ) | 108 | 110 | 3.625 | 4.62 - 400 | 0.9994 | 4.62 | 15.4 | 47.2 ± 11.3 | 94.3 | 11.3 |
| (1,4-NQ) | 130 | 158 | 7.941 | 1.43 - 100 | 0.9999 | 1.43 | 4.78 | 72.5 ± 1.4 | 145 | 1.44 |
| (9,10-AQ) | 208 | 180 | 12.437 | 0.58 - 100 | 0.9999 | 0.58 | 1.95 | 66.5 ± 1.0 | 133 | 0.99 |
| (9,10-PQ) | 180 | 208 | 16.049 | 11.8 - 100 | 0.9988 | 11.8 | 39.4 | 69.6 ± 0.6 | 139 | 0.63 |
|  |  |  |  |  |  |  |  | Added 100 ng mL^-1^ |  |  |
| (BA) | 230 | 202 | 21.580 | 2.20 - 100 | 0.9967 | 2.20 | 7.20 | 114 ± 9.3 | 114 | 9.33 |
|  |  |  |  |  |  |  |  |  |  |  |
|  |  |  |  |  |  |  |  | Added 500 ng mL^-1^ |  |  |
| (1,2-NQ) | 158 | 130 | 6.746 | 432 – 30,000 | 0.9994 | 432 | 1439 | 640 ± 0.9 | 128 | 0.89 |
|  |  |  |  |  |  |  |  |  |  |  |
| Nitro-PAHs | Ion# 1* | Ion #2** | RT |  |  | LOD | LOQ | Added 50 ng mL^-1^ |  |  |
|  | (m/z) | (m/z) | (min) |  |  | (pg) | (pg) | Recovered (ng mL^-1^) | Rec (%)  (n=3) | RSD(%) (n=3) |
| (1-NNAP) | 173 | 127 | 8.025 | 2.68 - 80 | 0.9975 | 2.68 | 8.92 | 46.2 ± 1.83 | 92.4 | 3.96 |
| (1M-4NNAP) | 187 | 115 | 8.182 | 3.83 - 60 | 0.9933 | 3.83 | 12.8 | 55.5 ± 1.22 | 111 | 2.20 |
| (2-NNAP) | 173 | 127 | 8.341 | 1.69 - 58 | 0.9981 | 1.69 | 5.61 | 54.7 ± 1.72 | 109 | 3.14 |
| (2-NBP) | 199 | 152 | 8.662 | 2.19 - 112 | 0.9989 | 2.19 | 7.28 | 53.7 ± 3.85 | 107 | 7.18 |
| (1M-5NNAP) | 187 | 115 | 8.926 | 3.02 - 72 | 0.9964 | 3.02 | 10.1 | 52.8 ± 1.16 | 106 | 2.19 |
| (1M-6NNAP) | 187 | 115 | 9.387 | 3.65 - 59 | 0.9924 | 3.65 | 12.2 | 51.3 ± 1.47 | 103 | 2.87 |
| (2M-4NNAP) | 187 | 115 | 9.691 | 3.83 - 73 | 0.9983 | 3.83 | 12.8 | 48.9 ± 0.25 | 97.8 | 0.52 |
| (3-NBP) | 199 | 152 | 10.153 | 2.14 - 79 | 0.9985 | 2.14 | 7.14 | 51.6 ± 1.62 | 103 | 3.15 |
| (4-NBP) | 199 | 152 | 10.599 | 2.39 - 69 | 0.9976 | 2.39 | 7.96 | 51.9 ± 1.54 | 104 | 2.97 |
| (5-NACE) | 199 | 152 | 13.230 | 1.59 - 174 | 0.9980 | 1.59 | 5.29 | 58.8 ± 0.44 | 118 | 0.75 |
| (2-NFLU) | 211 | 165 | 15.031 | 0.81 - 141 | 0.9992 | 0.81 | 2.70 | 59.7 ± 2.10 | 119 | 3.36 |
| (2-NPHE) | 223 | 165 | 15.493 | 0.93 - 143 | 0.9995 | 0.93 | 3.11 | 24.6 ± 2.09 | 81.9 | 8.50 |
| (3-NPHE) | 223 | 165 | 16.851 | 15.2 - 67 | 0.9943 | 15.2 | 50.7 | 24.1 ± 2.09 | 80.3 | 8.66 |
| (9-NPHE) | 223 | 165 | 17.716 | 1.53 - 174 | 0.9958 | 1.53 | 5.10 | 23.9 ± 1.98 | 79.5 | 8.28 |
| (2-NANT) | 223 | 176 | 18.172 | 10.1 - 149 | 0.9940 | 10.1 | 33.5 | 23.3 ± 2.02 | 77.7 | 8.66 |
| (9-NANT) | 223 | 176 | 18.553 | 13.0 - 70 | 0.9979 | 13.0 | 43.2 | 22.9 ± 1.69 | 76.4 | 7.37 |
| (2-NFLT) | 247 | 101 | 22.504 | 13.5 - 198 | 0.9809 | 13.5 | 46.1 | 23.2 ± 1.81 | 77.2 | 7.81 |
| (3-NFLT) | 247 | 101 | 22.553 | 8.08 - 61 | 0.9916 | 8.08 | 26.9 | 22.6 ± 1.89 | 75.5 | 8.36 |
| (1-NPYR) | 247 | 101 | 22.798 | 8.40 - 80 | 0.9980 | 8.40 | 28.0 | 22.7 ± 1.91 | 75.6 | 8.41 |
| (2-NPYR) | 247 | 101 | 23.439 | 10.7 - 186 | 0.9966 | 10.7 | 35.6 | 22.1 ± 2.00 | 73.6 | 9.04 |
| (4-NPYR) | 247 | 101 | 23.779 | 14.2 - 190 | 0.9926 | 14.2 | 47.3 | 22.0 ± 2.01 | 73.4 | 9.11 |
| (7-NBaA) | 215 | 273 | 26.340 | 15.1 - 74 | 0.9927 | 15.1 | 50.4 | 22.8 ± 1.84 | 75.8 | 8.09 |
| (6-NCRY) | 215 | 273 | 26.625 | 5.18 - 89 | 0.9933 | 5.18 | 17.2 | 23.9 ± 1.98 | 79.6 | 8.29 |
| (3-NBA) | 275 | 245 | 27.689 | 1.88 - 440 | 0.9983 | 1.88 | 6.27 | 25.7 ± 1.66 | 85.5 | 6.48 |
| (6-NBaP) | 297 | 267 | 31.731 | 8.89 - 150 | 0.9946 | 8.89 | 29.6 | 21.2 ± 1.66 | 70.6 | 7.83 |
| (1-NBeP) | 297 | 267 | 31.936 | 6.16 - 152 | 0.9980 | 6.16 | 20.5 | 21.7 ± 1.92 | 72.2 | 8.88 |
| (3-NBeP) | 297 | 267 | 33.509 | 9.14 - 100 | 0.9946 | 9.14 | 30.5 | 30.8 ± 3.56 | 103 | 11.55 |

*Quantification ion. **Identification ion


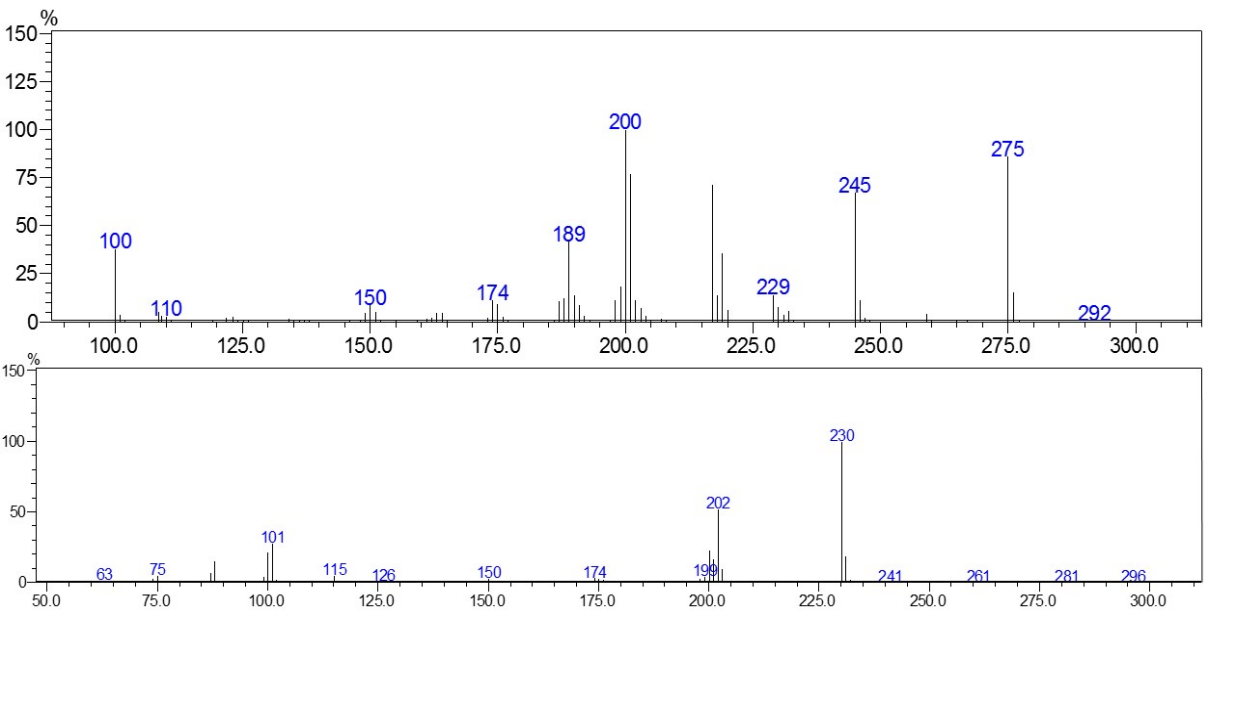

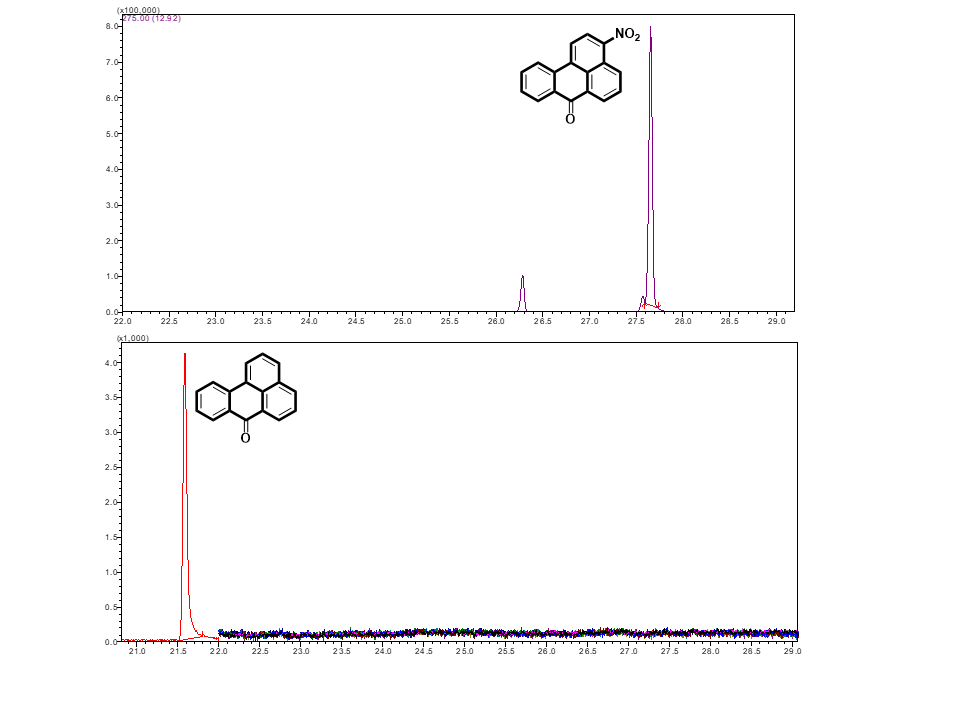
Figure S1. A) GC-MS chromatogram in Scan mode showing the well-defined peak of 3-NBA eluting in 27.58 min and B) BA eluting in 21.58 min. C) Mass spectrum obtaining from 3-NBA fragmentation in electron impact mode (EI). D) BA mass spectrum showing characteristic ions *m/z* 230 (ion base), 202, 200 and 101 (reference ions).

**C**

**D**

**C**

**D**

**B**

**A**


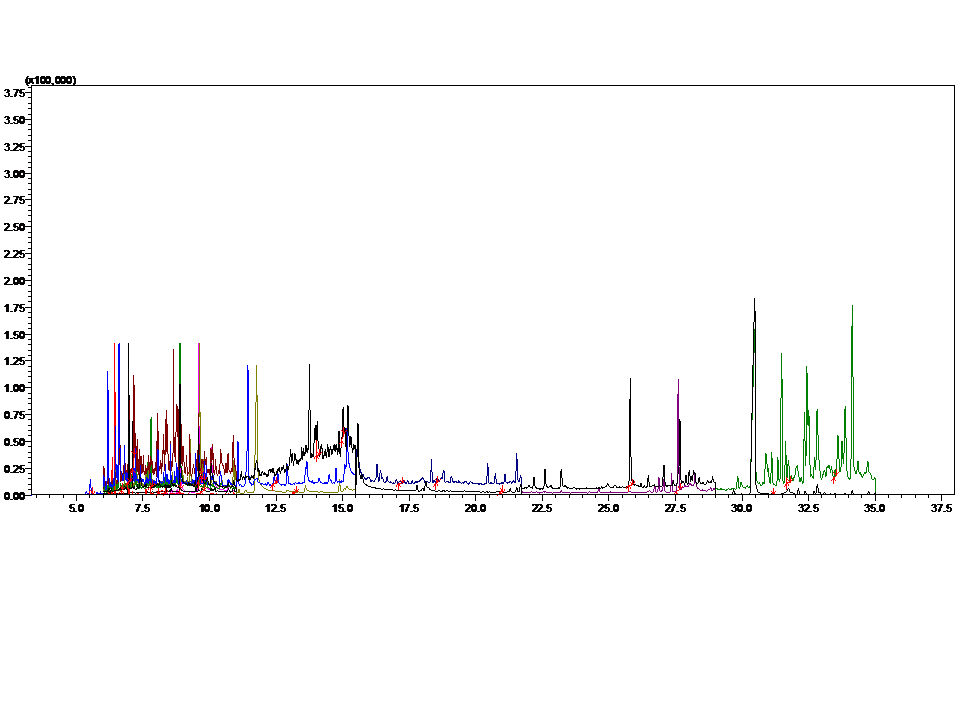
Figure S2. General chromatogram in SIM mode of a real polychaete sample collected in Madre de Deus site. Each compound is shown in detail in Figures S4 and S5.


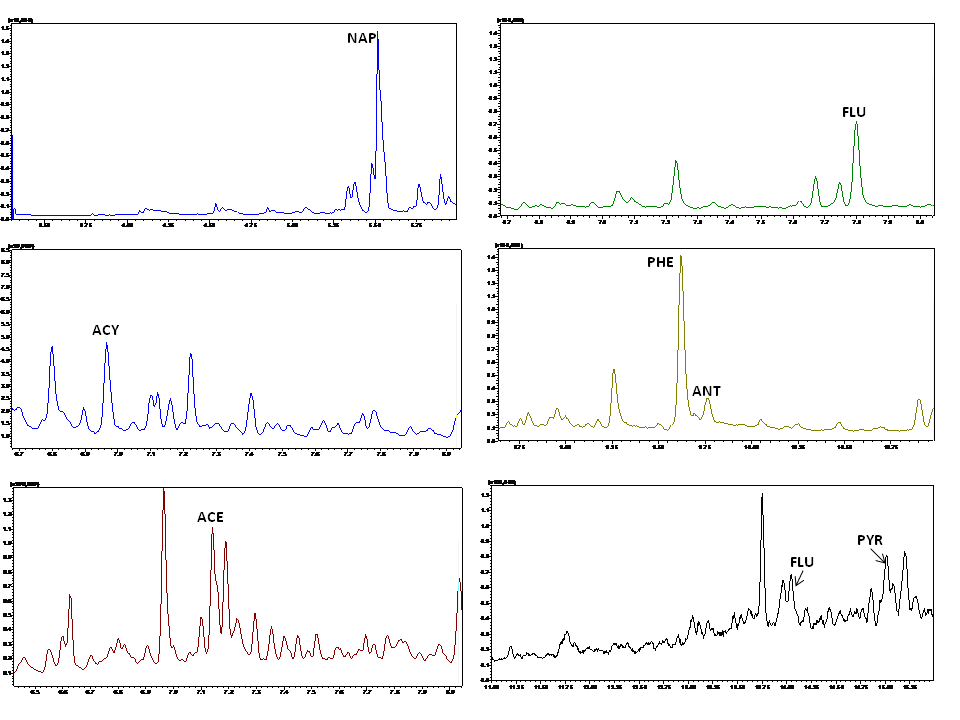

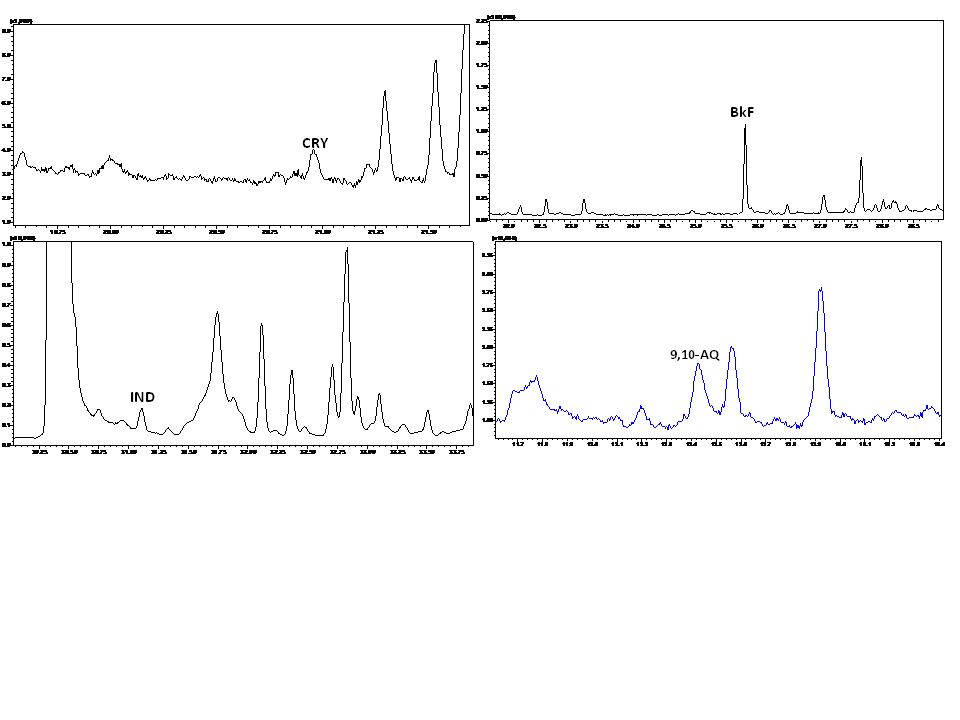


Figure S3. Real sample SIM chromatogram of detected PAHs and quinones in the Madre de Deus site. The compound were NAP (TR: 5.516 min), ACY (TR: 6.966 min), ACE (TR: 7.142 min), FLU (TR: 7.798 min), PHE (TR: 9.621 min), ANT (TR: 9.762 min), FLT (TR: 14.045 min), PYR (TR: 15.014 min), CRY (TR: 20.952 min), BkF (TR: 25.803 min), IND (TR: 31.113 min) and 9,10-AQ (TR: 12.427 min).


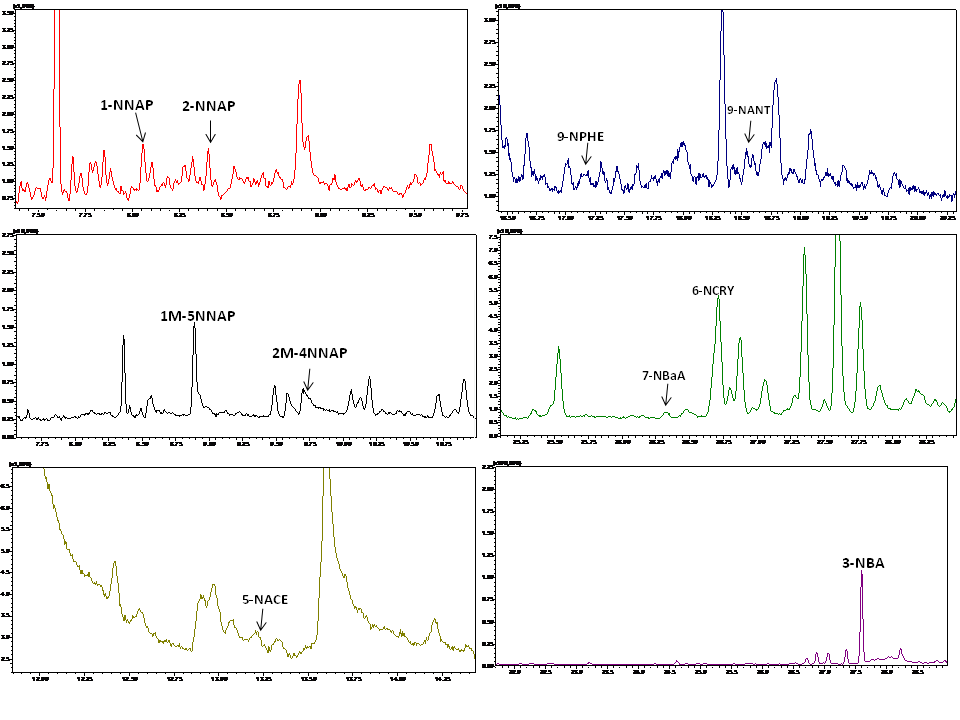

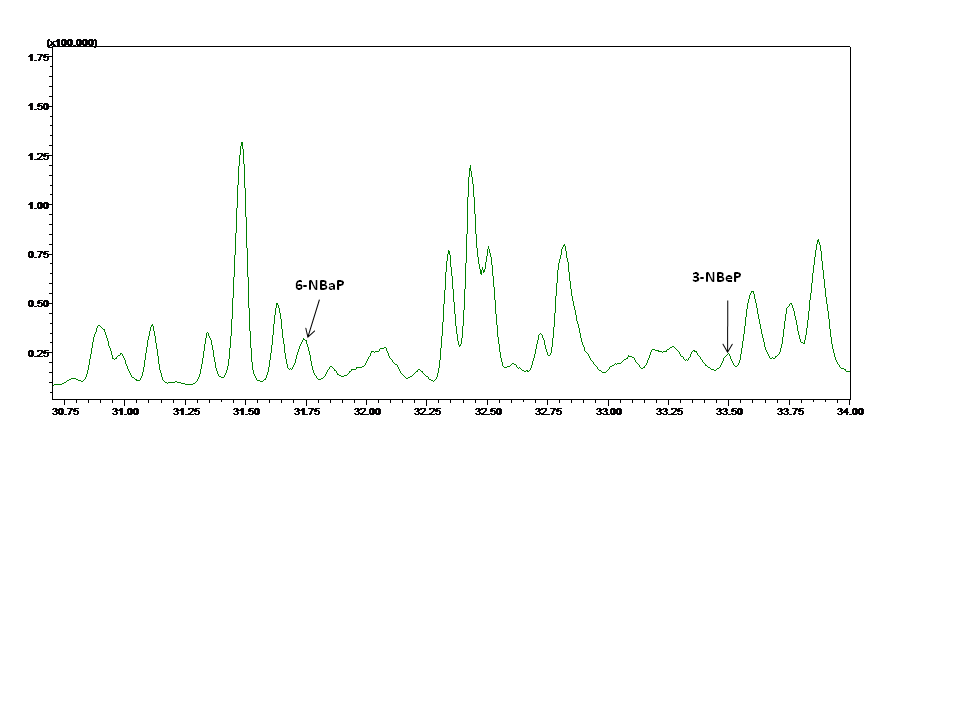
Figure S4. Chromatogram of nitro-PAHs detected in Madre de Deus sample. The compound were 1-NNAP (TR: 8.057 min), 2-NNAP (TR: 8.320 min), 1M-5NNAP (TR: 8.889 min), 2 M-4NNAP (TR: 9.700 min), 5-NACE (TR: 13.216 min), 9-PHE (TR: 17.190 min), 9-NANT (TR: 18.539 min), 7-NBaA (TR: 26.333 min), 6-NCRY (TR: 26.713 min), 3-NBA (TR: 27.61 min), 6-NBaP (TR: 31.731 min) and 3-NBeP (TR: 33.498 min).


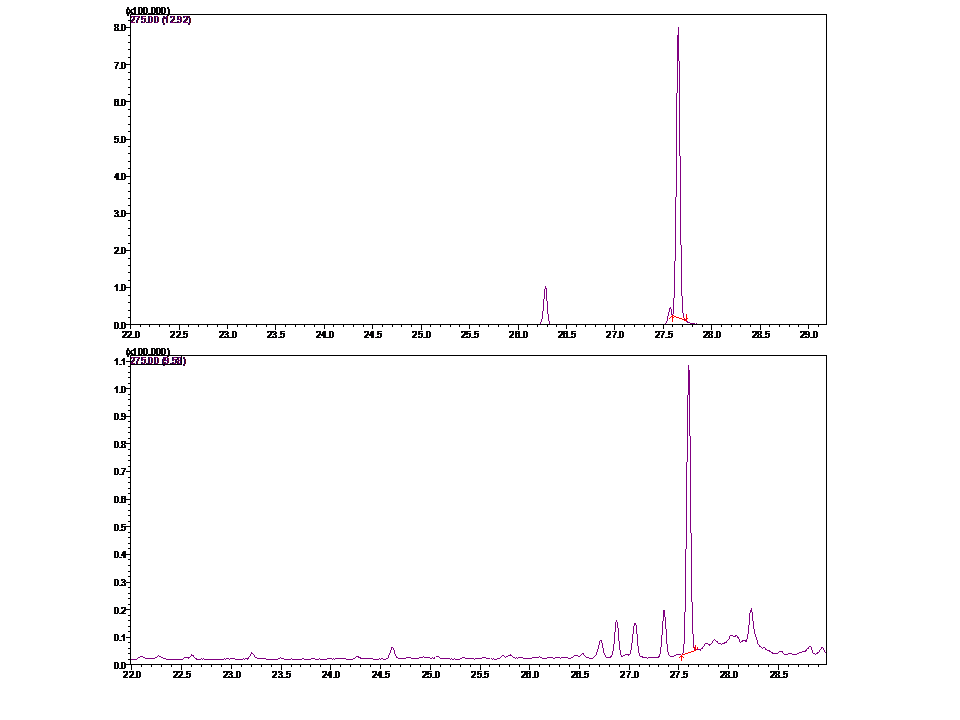

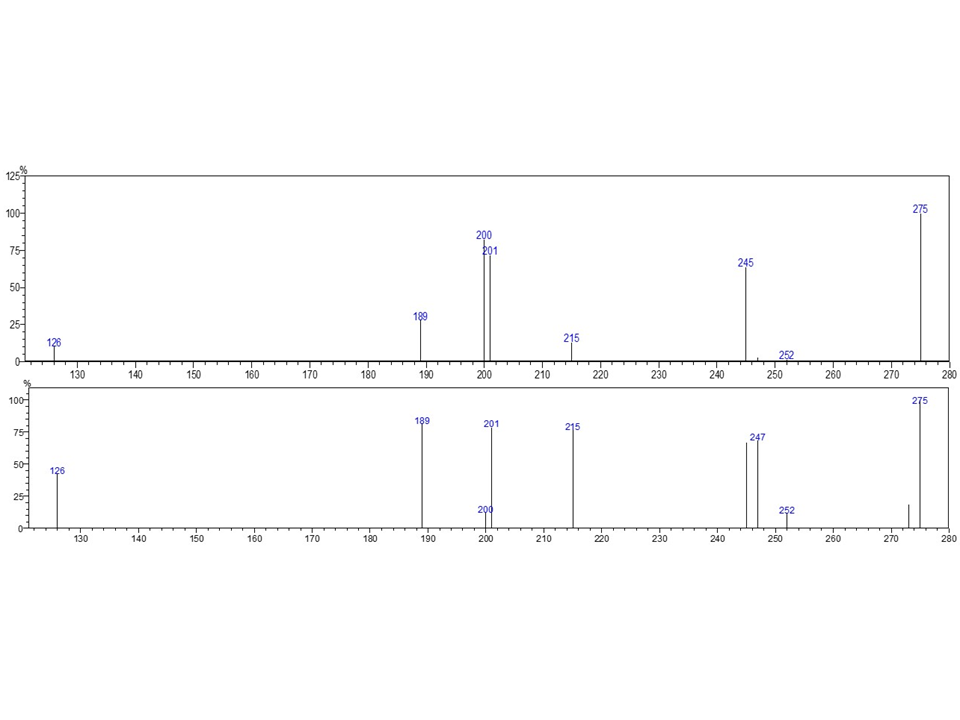
Figure S5. A) GC-MS standard solution chromatogram acquired in SIM mode with the 3-NBA peak. B) Chromatogram in SIM mode showing 3-NBA peak detected in a polychaete sample. C) Mass spectrum of the 3-NBA in the same standard solution showing the characteristic ions *m/z* 275 (M+ and base ion) and *m/z* 245. D) Mass spectrum in SIM mode of 3-NBA detected in a real sample collected in the BTS site.

**D**

**A**

**B**

**C**

**D**


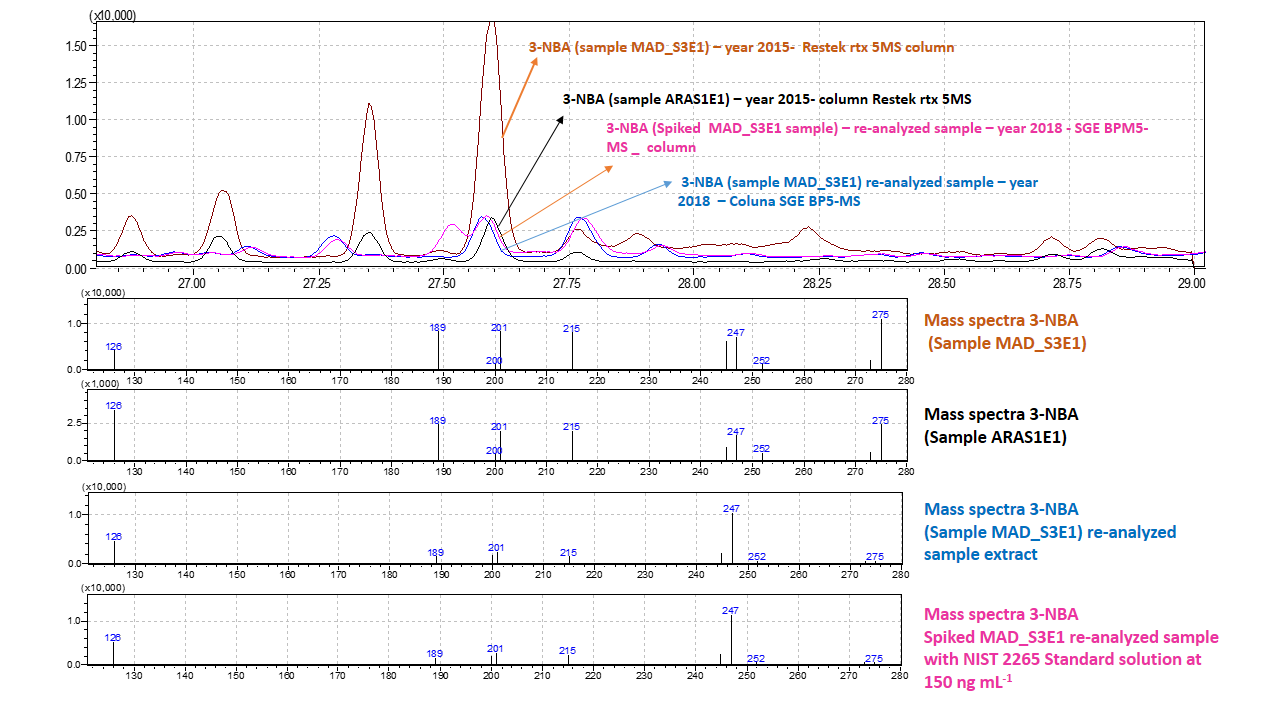
.

Figure S6. Comparison of different chromatogram for 3-NBA detected in different samples and spiked samples. The peaks were overlapped in order to facilitate the visualization of different samples.

**A**

.

**C**


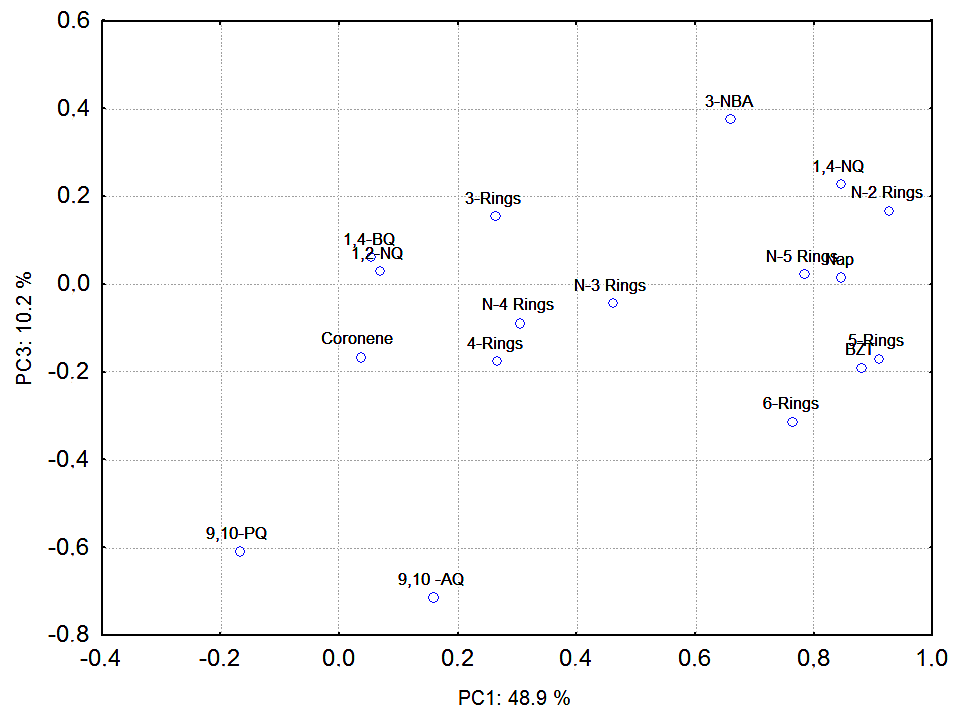

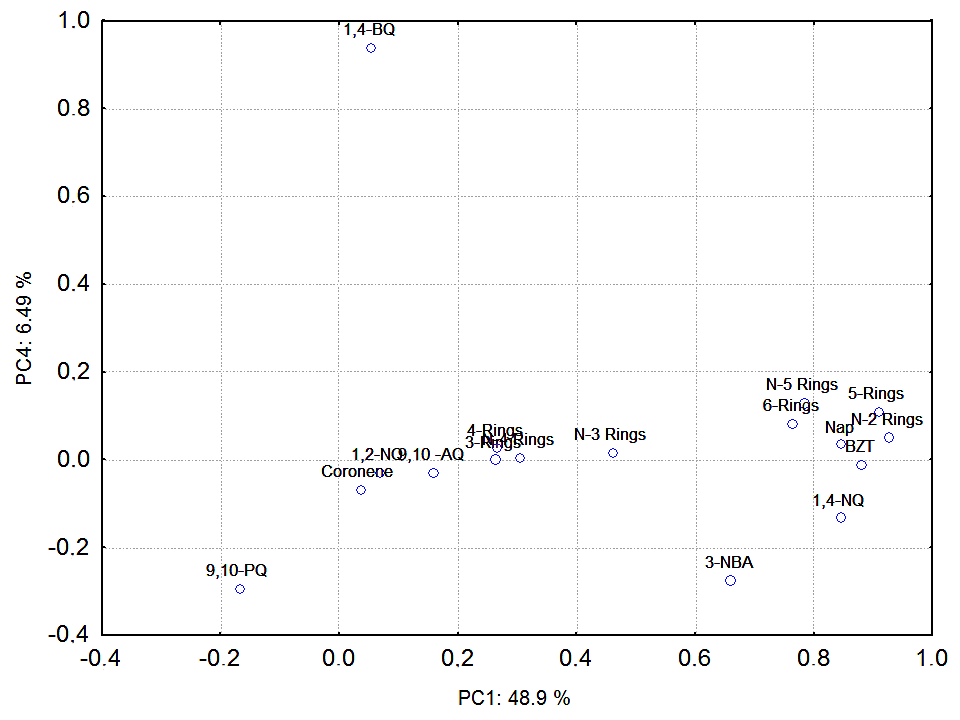

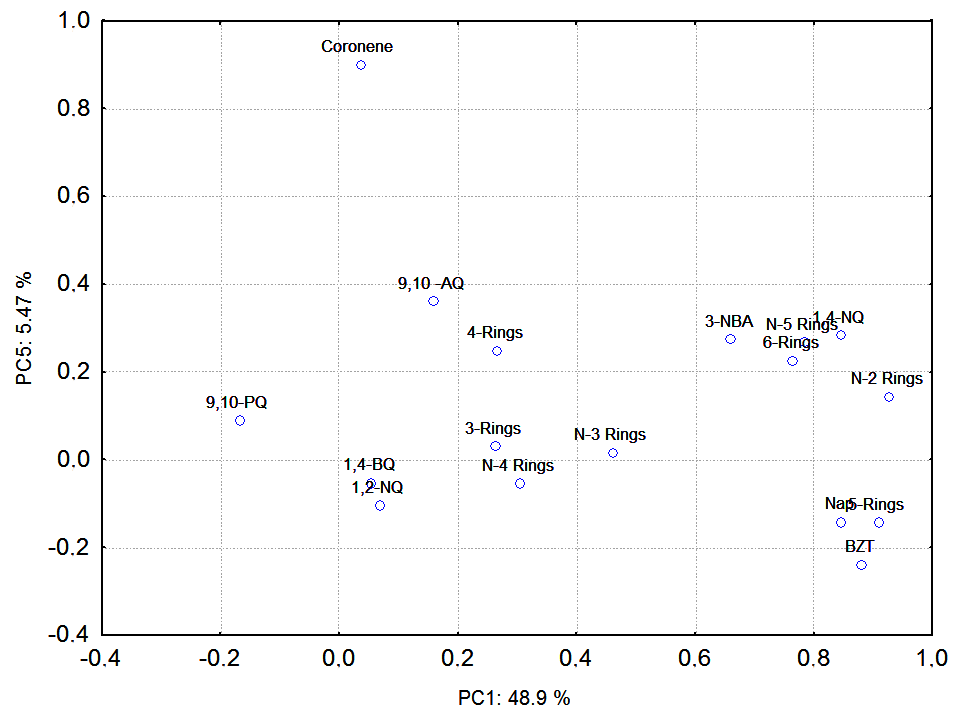

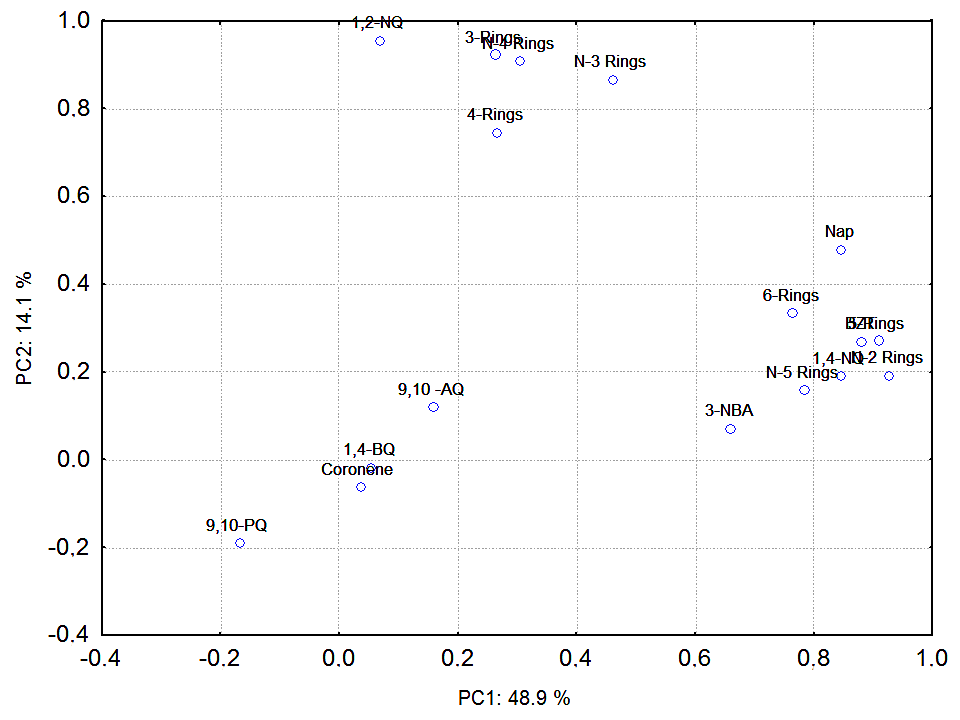
Figure S7. Score graphs obtained from principal component analysis. A) PC1xPC2 with approximately 63 % of total variance, followed by B) PC1x`PC3, 59 %, C) PC1xPC4, 55 % and D) PC1xPC5, 54 %.

**A**

**B**

**C**

**D**


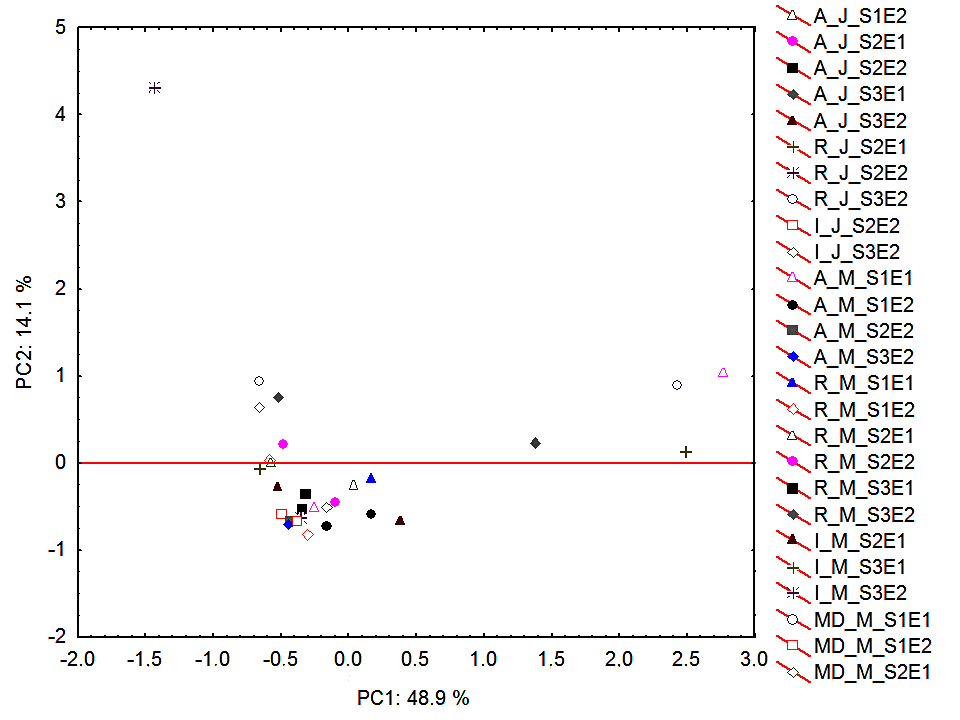

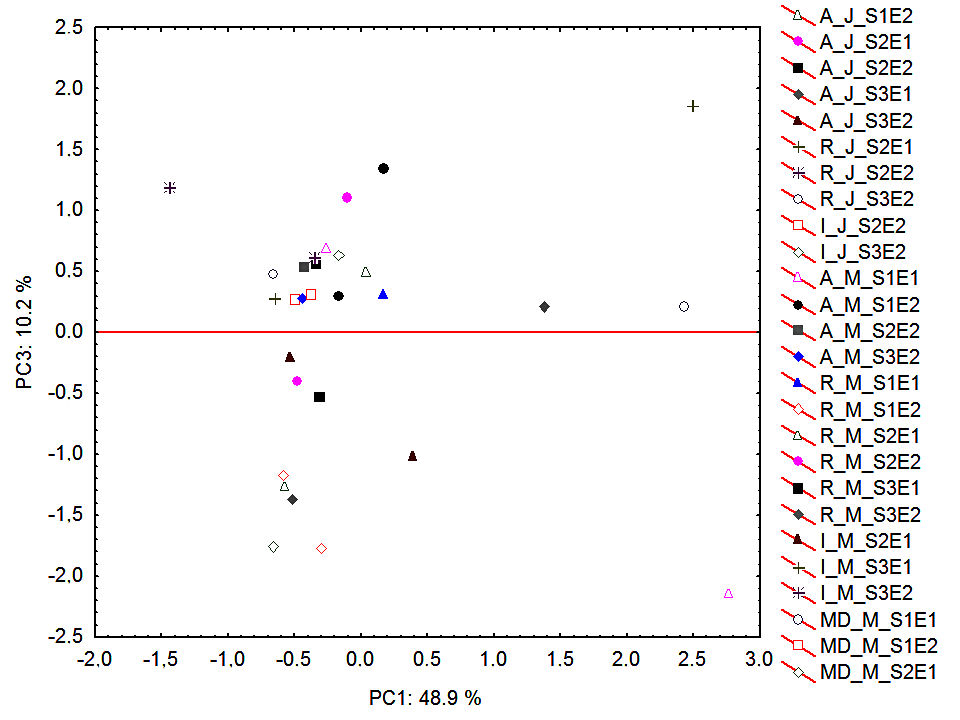


A

B


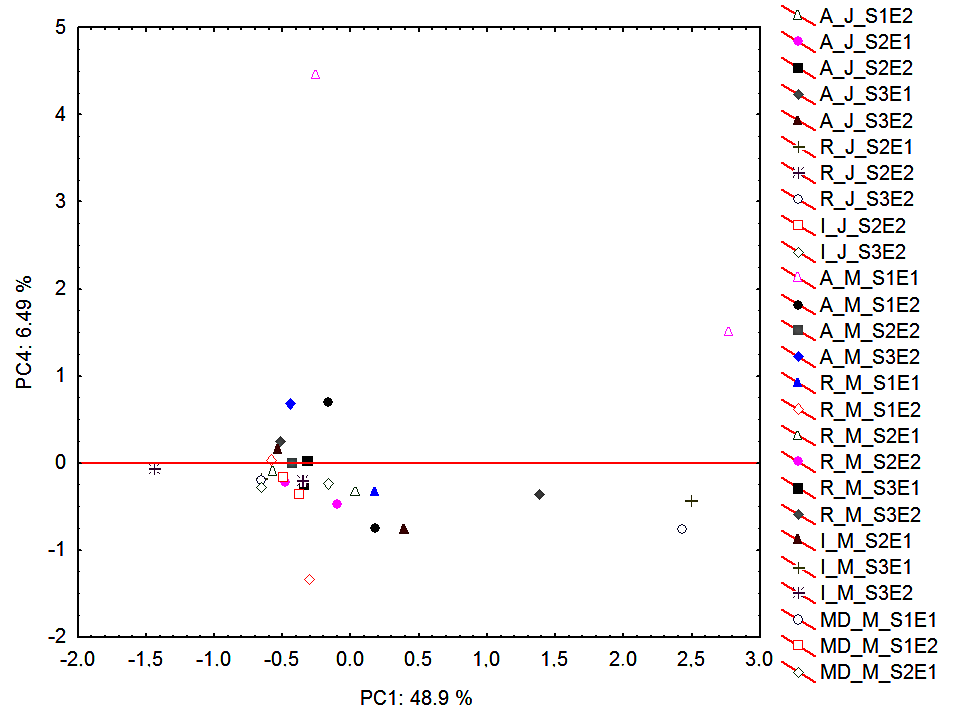

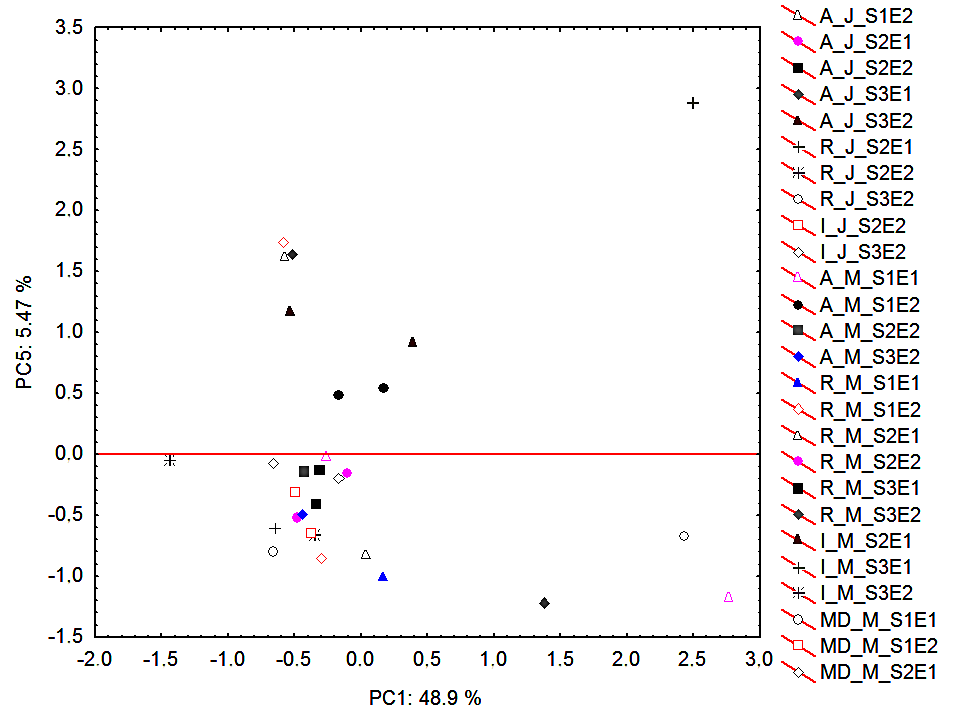
.

C

D

Figure S8. Loading graphs obtained from principal component analysis showing the distribution of polycheaetes samples. A) PC1xPC2 explaining approximately 63 % of total variance, followed by B) PC1x`PC3, 59 %, C) PC1xPC4, 55 % and D) PC1xPC5, 54 %. As can be seen in the loading graphs in Figure 1, the samples I_M_S3E1, MD_M_S1E1, A_M_S1E1 and A_J_S3E1 were discriminated in PC1 due to their high concentrations of naphthalene, 5 and 6-rings PAHs, nitro-PAHs containing 2 and 5 rings and oxy-PAHs such as 1,4-NQ and BA. As 3-NBA also presented high loading in this PC, these samples were characterized by high content of this compound. It is worth mentioning that these samples were collected throughout BTS sites corresponding to Inema, Madre de Deus, and Aratu sites. The samples collected in the Ribeira site (R_J_S2E2 and R_J_S3E2) were separated in PC2 and characterized by high concentrations of PAHs and nitro-PAHs containing 3 and 4 rings and oxy-PAHs such as 1,2-NQ. Although very little information is extracted from the last PCs, some oxy-PAHs such as 1,4-BQ and 9,10-AQ, and high molecular weight PAH like coronene (predominant in this PCs) were found in samples from Ribeira (R_J_S3E1, R_J_S2E2, R_M_S1E2, and R_M_S2E1), Aratu (A_M_S1E2, A_M_S1E1, and A_J_S3E1), and Inema (I_M_S3E1).
